# A novel injectable boron doped-mesoporous nano bioactive glass loaded-alginate composite hydrogel as a pulpotomy filling biomaterial for dentin regeneration

**DOI:** 10.1186/s12903-024-04808-3

**Published:** 2024-09-14

**Authors:** Marwa S. Naga, Hala M. Helal, Elbadawy A. Kamoun, Maha Abdel Moaty, Samia S. Abdel Rehim Omar, Ahmed Z. Ghareeb, Esmail M. El-Fakharany, Mona Mohy El Din

**Affiliations:** 1 Dental Biomaterials Department, Faculty of Dentistry, Alexandria University, Alexandria, Egypt; 2 Industrial Pharmacy Department, Faculty of Pharmacy, Alexandria University, Alexandria, Egypt; 3 Department of Chemistry, College of Science, King Faisal University, Al-Ahsa, 31982 Saudi Arabia; 4 Polymeric Materials Research Department, Advanced Technology and New Materials Research Institute (ATNMRI), City of Scientific Research and Technological Applications (SRTA-City), New Borg Al-Arab, Alexandria 21934 Egypt; 5 Oral Biology Department, Faculty of Dentistry, Alexandria University, Alexandria, Egypt; 6 Centre of Excellence for Drug Preclinical Studies (CE-DPS), Pharmaceutical and Fermentation Industry Development Centre, City of Scientific Research and Technological Applications (SRTA-City), New Borg Al-Arab, Alexandria 21934 Egypt; 7 Protein Research Department, Genetic Engineering and Biotechnology Research Institute GEBRI, City for Scientific Research and Technology Applications, New Borg Al-Arab, Alexandria 21934 Egypt; 8 Pharmaceutical and Fermentation Industries Development Centre (PFIDC), The City of Scientific Research and Technological Applications (SRTA City), Borg Al-Arab, Alexandria Egypt

**Keywords:** Injectable scaffolds, Sodium alginate hydrogels, Mesoporous bioactive glass nanoparticles, Boron NPs, Regenerative pulpotomy, Dentin regeneration

## Abstract

**Background:**

Different materials have been used as wound dressings after vital pulp therapies. Some of them have limitations such as delayed setting, difficult administration, slight degree of cytotoxicity, crown discoloration and high cost. Therefore, to overcome these disadvantages, composite scaffolds have been used in regenerative dentistry. This study aims to construct and characterize the physicochemical behavior of a novel injectable alginate hydrogel loaded with different bioactive glass nanoparticles in various concentrations as a regenerative pulpotomy filling material.

**Methods:**

Alginate hydrogels were prepared by dissolving alginate powder in alcoholic distilled water containing mesoporous bioactive glass nanoparticles (MBG NPs) or boron-doped MBG NPs (BMBG NPs) at 10 and 20 wt% concentrations. The mixture was stirred and incubated overnight in a water bath at 50 ^0^ C to ensure complete solubility. A sterile dual-syringe system was used to mix the alginate solution with 20 wt% calcium chloride solution, forming the hydrogel upon extrusion. Then, constructed hydrogel specimens from all groups were characterized by FTIR, SEM, water uptake percentage (WA%), bioactivity and ion release, and cytotoxicity. Statistical analysis was done using *One-Way ANOVA* test for comparisons between groups, followed by multiple pairwise comparisons using Bonferroni adjusted significance level (*p* < 0.05).

**Results:**

Alginate/BMBG loaded groups exhibited remarkable increase in porosity and pore size diameter [IIB1 (168), IIB2 (183) (µm)]. Similarly, WA% increased (~ 800%) which was statistically significant (*p* < 0.05). Alginate/BMBG loaded groups exhibited the strongest bioactive capability displaying prominent clusters of hydroxyapatite precipitates on hydrogel surfaces. Ca/P ratio of precipitates in IIA2 and IIB1 (1.6) were like Ca/P ratio for stoichiometric pure hydroxyapatite (1.67). MTT assay data revealed that the cell viability % of human gingival fibroblast cells have declined with increasing the concentration of both powders and hydrogel extracts in all groups after 24 and 48 h but still higher than the accepted cell viability % of (˃70%).

**Conclusions:**

The outstanding laboratory performance of the injectable alginate/BMBGNPs (20 wt%) composite hydrogel suggested it as promising candidate for pulpotomy filling material potentially enhancing dentin regeneration in clinical applications.

**Supplementary Information:**

The online version contains supplementary material available at 10.1186/s12903-024-04808-3.

## Background

Maintaining pulp vitality is of great importance in restorative dentistry. Pulpotomy preserves pulp vitality, it helps radicular pulp to heal and create a dentin-like tissue seal [1]. Various medicaments such as, calcium hydroxide, formocresol, Mineral trioxide aggregate (MTA), Bio-dentine have been suggested to be applied in pulpotomy [2]. But recently none of them has proved to be ideal [3]. Such a gap was the directing stimulator behind the search for a biocompatible biomaterial that fulfills all requirements needed to achieve a successful regenerative pulpotomy procedure [3]. Therefore, to overcome these disadvantages, the regeneration of dental tissues using a scaffold-based tissue engineering strategy represents a promising approach to replace damaged dentin/pulp structures and restore their biological functions [4].

Injectable hydrogels are soft scaffolds that mimic extracellular matrix structure and are important in dentin pulp complex regeneration. They act as a template for delivery of therapeutic cells efficiently and bioactive molecules to target sites in a minimally invasive procedure [5].

Hydrogels made of natural polymers (alginate, gelatin, keratin etc.) have many advantages such as, being biocompatible, inexpensive and readily available [5]. Alginate is an anionic polysaccharide, derived from brown algae. It is cross-linked with divalent metal ion to produce water-soluble hydrogel [6]. Alginate hydrogel is biocompatible, nontoxic, biodegradable and non-immunogenic. Additionally, alginate has a good hydrogel forming characteristics under physiological conditions and high swelling ability. Alginate hydrogels provide a matrix to support dental regeneration and it has been proven that it up regulates dentine matrix secretion [7]. Bioactive glass nanoparticles (BG-NPs) were suggested and selected in dentin regeneration due to their positive effects such as, bioactivity, antibacterial, angiogenesis. Moreover, BG-NPs bind to living tissues, stimulate new tissue growth while dissolving over time and they offer more surface area to combine with biomaterials [8].

Recently, mesoporous BGs nanoparticles (MBG NPs) have been developed. They are fabricated in a way that will provide MBG NPs with higher specific surface area, highly ordered pores, excellent apatite forming ability, cell viability and bioactivity than conventional BGs [9]. MBG NPs enhance antimicrobial and tissue regenerative potential [9, 10]. They potentiate odontogenic differentiation of SHEDs and dentin-like matrix mineralization [11].

Boron (B) is the fifth element of the periodic table, has been presented in Group 13. It influences the performance of several metabolic enzymes. Addition of boron to BGs leads to increase in biodegradability, bioactivity and biocompatibility [12]. The combination of the abovementioned biomaterials provides a biomimetic approach suitable for dental tissue regeneration mixing the advantages of each individual material and minimizing their disadvantages.

Accordingly, the current study aims to formulate, optimize and characterize a novel injectable alginate composite hydrogel loaded with MBG NPs in various concentrations (10 and 20 wt%) as a promising regenerative pulpotomy filling material. The synthesized composite hydrogels have been assessed in terms of their physicochemical properties and cell viability.

## Materials and methods

### Materials

Mesoporous bioactive glass nanoparticles (58% SiO_2_, 33% CaO, and 9% P_2_O_5_) (NT-MBG 58S3.6), boron doped-mesoporous bioactive glass nano particles (44% SiO_2_, 33% CaO, and 9% P_2_O_5_, 14% boron) (NT-MBG 58S3.6 B14) were all acquired from NanoTech. Co. Egypt. Sodium alginate powder (medium viscosity), calcium chloride anhydrous powder (95%), ethanol (90%) and all reagents for simulated body fluid (SBF) (sodium chloride, sodium bicarbonate, potassium chloride, potassium phosphate dibasic trihydrate, 1 M hydrochloric acid solution, magnesium chloride hexahydrate, calcium chloride, sodium sulfate, Tris–hydroxymethyl aminomethane (TRIS)) were all supplied from Sigma-Aldrich Chemie, Germany. Human gingival specimens were obtained from the faculty of dentistry, Oral Surgery Department, Alexandria University, Egypt. Then human gingival fibroblast cells were isolated in the Center of Excellence for Research in Regenerative Medicine and Applications, Alexandria University, Egypt. MTT (3-(4,5-dimethylthiazol-2-yl)-2,5-diphenyltetrazoliumbromide) was acquired from (Thermo Fisher Scientific, Massachusetts, USA). Dimethylsulfoxide (DEMSO) was purchased from Sigma Chemicals Co. (St Louis, USA).

### Methods

In the laboratory controlled (in-vitro): A total number of 35 hydrogel discs were utilized for the characterization objective. Built on comparison of means, sample size was estimated as 6 for each group raised to 7 to balance out laboratory procedure errors [13]. Sample size was determined presuming alpha error = 5%, study power = 80% [14], and ascertained by *GPower 3.1.9.4* sample size calculator software.

Tested specimens were assigned into two major groups according to the loading of alginate matrix (hydrogel) with mesoporous bioactive glass nanoparticles (MBGNPs): Group I: unloaded sodium alginate hydrogel (control) (*n* = 7), group II: sodium alginate/MBG NPs (*n* = 28). Then group II was subdivided into two subgroups based on the modification of mesoporous bioactive glass (MBG NPs): Subgroup IIA: sodium alginate/mesoporous bioactive glass nanoparticles (NT–MBG 58S3.6) (MBG NPs) (*n* = 14) and subgroup IIB: sodium alginate/boron doped mesoporous bioactive glass nanoparticles (NT–MBG 44S3.6 B14) (BMBG NPs) (*n* = 14). Again subgroups IIA and IIB, each was further subdivided into two other subgroups according to the concentration of MBG NPs: subgroup IIA1:10 wt% NT–MBG 58S3.6 (*n* = 7), subgroup IIA2: 20 wt% NT–MBG 58S3.6 (*n* = 7), subgroup IIB1:10 wt% NT–MBG 44S3.6 B14 (*n* = 7) and subgroup IIB2 20 wt% NT–MBG 44S3.6 B14 (*n* = 7). All specimens were grouped using random allocation procedures [13, 14], (Tables 1 and 2).

**Table 1:** Nominal composition of mesoporous bioactive glass nanoparticles

Sample ID	Reactants (mole)			
	SiO_2_	CaO	*P*_2_O_5_	B_2_O_3_
NT-MBG 58S3.6 (MBG NPs)	58	33	9	0
NT-MBG 44S3.6 B14 (BMBG NPs)	44	33	9	14

**Table 2:** Experimental groups composition used for preparing the injectable composite hydrogels

Groups/Subgroups	Sodium alginate matrix (Na-Alg: CaCl_2_)	Bioactive glass nanoparticles percentage (wt%)
I	7:20	0
IIA1	7:20	10
IIA2	7:20	20
IIB1	7:20	10
IIB2	7:20	20

## Methodology

### Synthesis of injectable sodium alginate hydrogel

For determination of the optimum criteria for synthesizing the injectable sodium alginate hydrogel: The optimum condition was found out at 7 wt% sodium alginate and 20 wt% calcium chloride anhydrous were chosen for constructing the injectable sodium alginate hydrogel which exhibited the best setting time at (15 min), injectability (easy-moderate), viscosity measurements, pH values, swelling ratio percentage at preset time points and hydrolytic degradation (weight loss percentage).

**Fig. 1:**
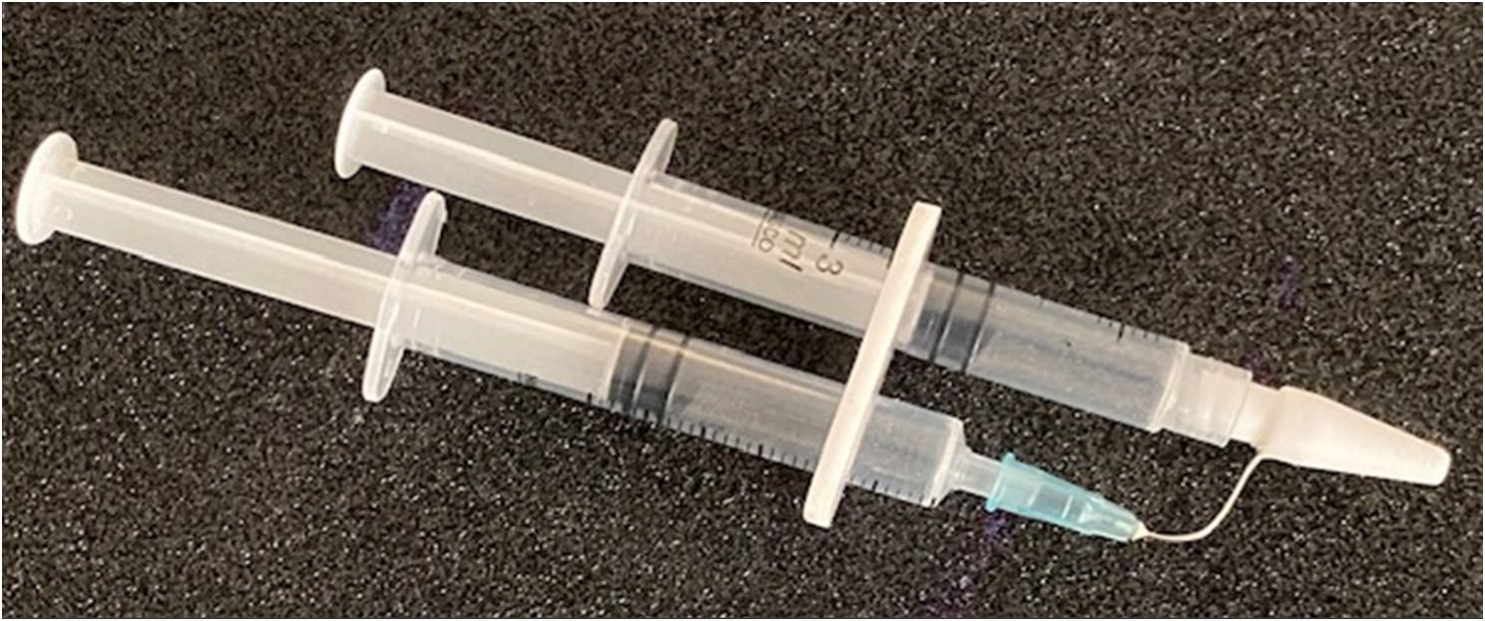
Assembled dual- syringe system

Typically, 7 wt% aqueous sodium alginate phase was acquired by dissolving, homogenously, sodium alginate powder in sterile distilled water under aseptic conditions, using magnetic stirrer until a complete clear solution prevailed then the solution was filtered using a sterile syringe filter. Then, mineral phase (20 wt% calcium chloride anhydrous) was attained by dissipating calcium chloride anhydrous powder in sterile distilled water [15, 16]. Finally, both phases were then packed into separate chambers of a specific assembled sterile dual-syringe system for merging and injection resulting in ejection of a homogeneous formulation [17] (Fig. 1).

### Preparation of injectable different mesoporous bioactive glass nanoparticles (MBG NPs, BMBG NPs) loaded-sodium alginate composite hydrogel

Same laboratory steps were performed for loading the formulated sodium alginate hydrogel with different MBG NPs in various concentrations (10 and 20 wt%) as illustrated in Fig. S1 (*Supplementary data*).

#### Laboratory characterization of the novel injectable alginate hydrogel scaffolds

For laboratory characterization hydrogel discs (10 mm in diameter × 5 mm in height) were constructed for each group. The hydrogel was injected into specially assembled Teflon molds (10 mm diameter, 5 mm height), to obtain a standardized hydrogel disc configuration.

### Instrumental characterization

#### FTIR analysis

A total of 7 discs (dimensions 10 mm diameter× 5 mm height) for fabricated scaffolds in each group were prepared for chemical bond structure evaluation using Fourier-transform Infrared Spectroscopy (FTIR) model: (Spectrum 100; Perkin Elmer, Germany) at wavenumber 400–4000 cm^− 1^ and resolution 2 cm^− 1^ [18, 19].

#### SEM and EDX investigation

Surface morphology and porosity of the constructed sodium alginate composite hydrogels specimens were assessed by scanning electron microscope (SEM). A total of 7 hydrogel discs of dimensions (10 mm diameter × 5 mm height) from each test group were made for evaluating surface morphology using SEM (JSM-IT200; JEOL, Japan) [19, 20]. Furthermore, the elemental composition of the precipitated apatite layer on top of the hydrogel discs was investigated via energy dispersive X-ray spectroscopy (EDX) (JSM-IT200; JEOL) at magnification = x 1500 and cps/ev = 784/130 [19]. Calcium to phosphate ratio (Ca/P) of each specimen was evaluated.

### Physicochemical measurements

#### Water uptake (%)

Hydrogel discs (*n* = 7) (10 mm diameter ×5 mm height) from each study group were made ready to measure water uptake (%) based on the equilibrium swelling theory state of hydrogels. The hydrogel discs were submerged in distilled water at room temperature. Then discs were weighed using sensitive balance at predetermined time intervals with increasing hydrogel disc’s weight and volume because of swelling process. Weighing was repeated until there was no change in hydrogel disc’s weight (Equilibrium swelling state). Specimens were then withdrawn, and excess moisture was removed [21].

The water uptake (%) of hydrogels was calculated as follows:

1 $$Water\,uptake\,\left( \% \right) = \left[ {\left( {{W_s} - {W_0}} \right)/{W_0}} \right] \times 100$$.

Where, W_s_ is the weight of swollen hydrogel and W_0_ is the weight of dry hydrogel.

#### In vitro bioactivity and ions release

In vitro bioactivity and ions release were studied for the sodium alginate hydrogel before and after loading with the BG NPs. Hydrogel discs (10 mm in diameter × 2 mm in height) from each selected group were submerged in sealed polyethylene falcon tubes containing simulated body fluid (SBF) solution (22 mL) [22] and incubated at 37oC in a shaking incubator (90 rpm) for 1, 3, 7, 14, and 21 days. At each time point, SBF solution was collected, specimens were carefully washed with deionized water, then dried followed by measurement of weight variation [19], FTIR [19] analysis, SEM – EDX [19], and In vitro ions release and pH of SBF medium [23, 24].

#### In vitro- bioactivity evaluation

##### Weight variation

The obtained weight gain determined the rate of the conversion (bioactivity) which was calculated as given equation:

2 $$W\% = \left[ {\left( {{W_0} - {\rm{ }}{W_{f }}} \right)/{W_0}} \right] \times 100$$.

Where, W_0_ is the initial weight of the material and W_f_ is the final weight [19].

##### FTIR, SEM and EDX analyses

This part has been mentioned above in the section of instrumental characterization.

#### In vitro ion release and pH evaluation

At each time point, supernatant samples of the collected SBF solution were tested using inductively coupled plasma-mass spectroscopy (ICP-MS) (iCAP, Thermo, Germany) according to methods mentioned in APHA (American Public Health Association), (2017) [24], to determine the concentrations of the solute ions (Calcium, phosphorous, boron, and silicon) to evaluate the degree of bioactivity of the hydrogel material. The recovery of metals was within the certified limits as 10 ppb to 1000 ppb. To get the final concentration, the solution was diluted with 0.1 mM H_2_NO_3,_ and the final dilution factor was used. *Qtegra* software (USA) was used for average and relative standard deviation calculations. Additionally, the pH of the SBF medium in which specimens were placed was also measured using a digital pH meter [23].

### Cell viability (%) by MTT assay

The current study was accepted by the Institutional Ethical Committee, Faculty of Dentistry, Alexandria University (IRB No. 00010556-IORG No. 0008839) (0240-April 2021). Cell viability assessment was performed by indirect method in compliance with (ISO 10993-5) specifications. The effect of BG NPs nanoparticles and hydrogel extracts on cell viability % was evaluated using MTT cell viability assay. Yellow, water soluble MTT (3-(4,5-dimethylthiazol-2-yl)-2,5-diphenyltetrazoliumbromide was metabolically reduced to blue-violet insoluble formazan in viable cells. Human gingival specimens were obtained from the Faculty of Dentistry, Oral Surgery Department, Alexandria University. Then all steps of isolation of the gingival cells were performed in the Center of Excellence for Research in Regenerative Medicine and Applications, Alexandria University. Human gingival fibroblast cells were acquired from an earlier formed cell line cultured from healthy patients. Gingival fibroblasts of the third to fifth passage were utilized in this study. Then fibroblast suspension was seeded into sterile flat bottom microplates and cultured then incubated in an incubator for 24 h at 37^o^C in 5% CO_2_ for one day. Next, different concentrations of nanoparticles and hydrogels extracts from all study groups were added to the cells and incubated at 37^o^C in 5% CO_2_. After 24 and 48 h all extracts were taken out and cells were rinsed with phosphate buffered saline solution (PBS) to eliminate dead cells and any debris. 200 µL of MTT solution (0.5 mg/mL) were put on each well and incubated at 37^o^C in 5% CO_2_ for 5 h. The absorbance (A) was determined at 630 nm using microplate ELISA reader (Enzyme linked immunosorbent assay) after the disappearance of the purple formazan crystals in 200 µL/well of DMSO. The relative cell viability (%) in comparison to control wells including cells without putting in extracts were measured using the following Eqs. [25, 26]:

3 $$Cell\,viability\,\left( \% \right) = \left( {{A_{test}}} \right)/({A_{control}}) \times 100$$

Where, A _test_ is cells number after incubation and A _control_ is initial cells number before incubation.

### Statistical analysis

Data were analyzed using IBM SPSS for windows (*Version 23.0*) and significance was set at p value < 0.05. Means and standard deviations (SD) were calculated for all variables. *One-Way ANOVA* test was used for comparisons between groups, followed by multiple pairwise comparisons using Bonferroni adjusted significance level.

## Results

### FTIR analysis of composite hydrogels

**Fig. 2:**
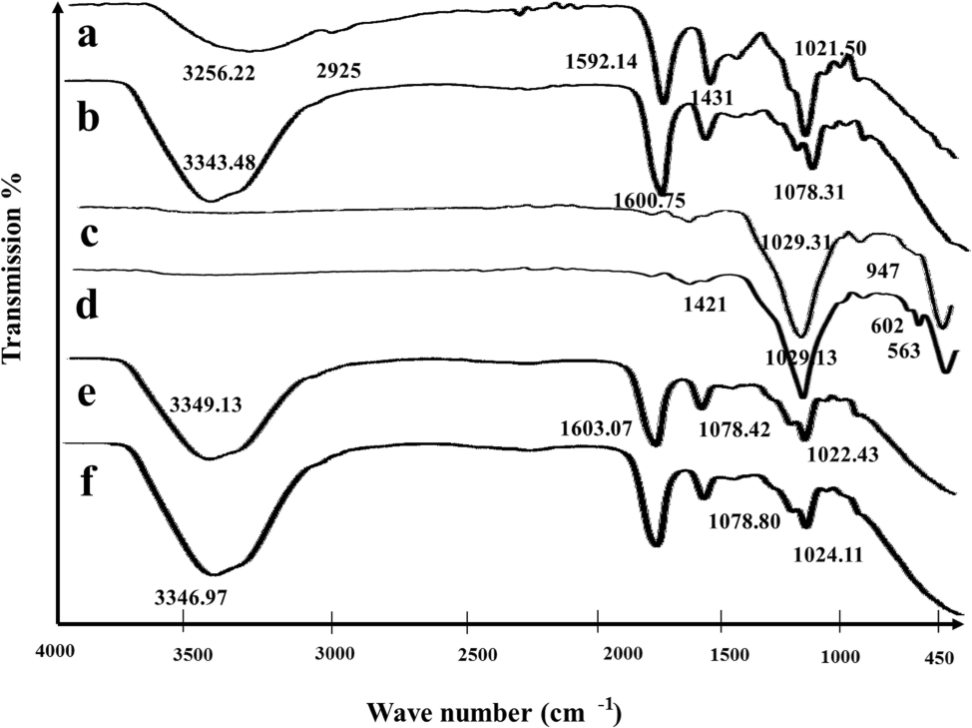
FTIR spectra of sodium alginate powder (**a**), crosslinked unloaded sodium alginate hydrogel (control) (**b**), mesoporous bioactive glass nanoparticles (MBG NPs) (powder) (**c**), boron doped-mesoporous bioactive glass nanoparticles (BMBG NPs) (powder) (**d**), crosslinked sodium alginate /MBG NPs loaded hydrogel (**e**), and crosslinked sodium alginate/BMBG NPs loaded hydrogel (**f**)

Figure 2 clearly exhibits the IR spectra of pure sodium alginate powder, different BG NPs, cross linked sodium alginate hydrogels before and after loading with the studied BG NPs. IR spectrum of sodium alginate powder shows a broad band from ν 3000 to 3500 cm^− 1^ which conferred with O-H stretching. The band at ν 2925 cm^− 1^ assigns to alkyl stretching mode (ν -CH) (Fig. 2a). IR spectrum of crosslinked unloaded sodium alginate hydrogel displays bands at ν 1600 and 1431 cm^− 1^ which attribute to stretching vibrations of -COOH (asymmetric) and -COOH (symmetric) in alginate; respectively. Specifically, these two bands (ν 1600 and 1431 cm^− 1^) are the most valuable distinctive bands to determine the ion crosslinking action. Further, a common characteristic peak related to alginate has been found at ν 1022 cm^− 1^ which is associated with C-O-C group (Fig. 2b). Representative IR spectrum of MBG NPs (NT -MBG 58S3.6) shows bands at ν 1029 and 947 cm^− 1^ which are associated with Si-O-Si asymmetric stretching and Si-NBO stretching; respectively (Fig. 2c). In IR spectrum of BMBG NPs (NT-MBG 44S3.6B14), bands at ν 1421, 602 and 563 cm^− 1^ are assigned to stretching vibrations of B-O bonds in [BO_3_] units and bending vibrations comparable to B-O-B bond; respectively (Fig. 2d). Illustrative IR spectrum of loaded hydrogel with NT-MBG 58S3.6 nanoparticles exhibit bands at approximately ν 1078 cm^− 1^ that are associated with Si-O-Si asymmetric stretching mode (Fig. 2e). It can be noted in IR spectra of both loaded alginate hydrogels the existence of the vibrational band at ν 1022 cm^− 1^ which contributed to silanol groups (Si-OH) often present in silica constructed by sol-gel technique. As well broad bands located in the range from ν 3000 to 3650 cm^− 1^ are related to both shares of hydroxyls (sodium alginate) and silanols (BG NPs) (Fig. 2e and f).

### SEM investigation of composite hydrogels

**Fig. 3:**
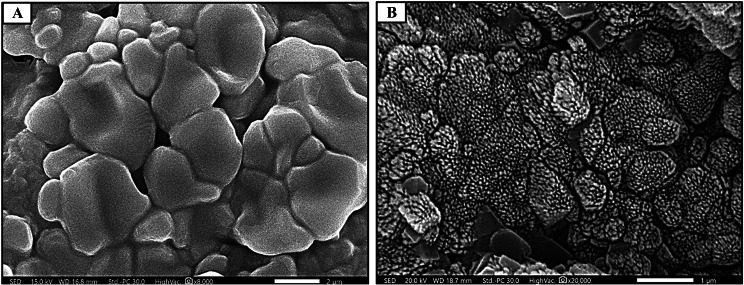
SEM images (surface view) of freeze dried crosslinked unloaded sodium alginate hydrogel (**A**), and mesoporous bioactive glass nanoparticles (MBG NPs) loaded-alginate composite hydrogel (**B**) (original magnification X8000, X20000)

**Fig. 4:**
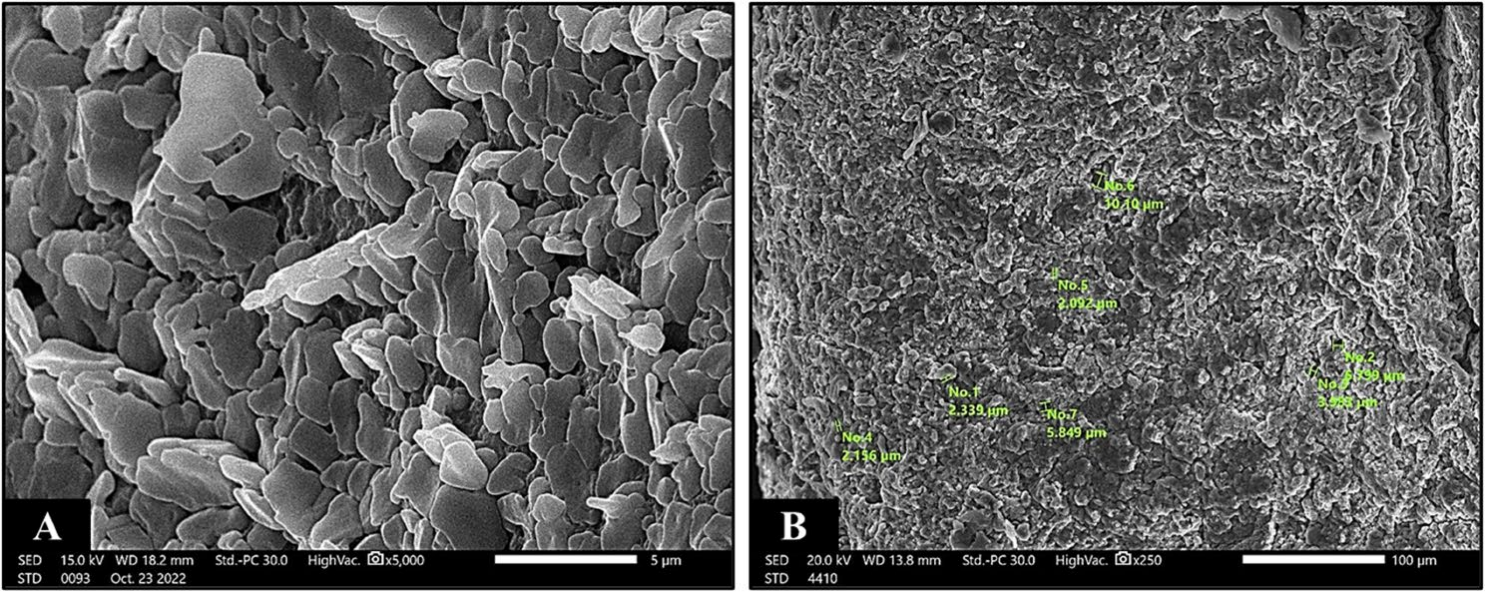
SEM images of freeze-dried cross-linked unloaded sodium alginate hydrogel (Control) revealing the nano size pore configuration (**A**) (surface view, original magnification X5000), (**B**) (cross-sectional view, original magnification X250)

**Fig. 5:**
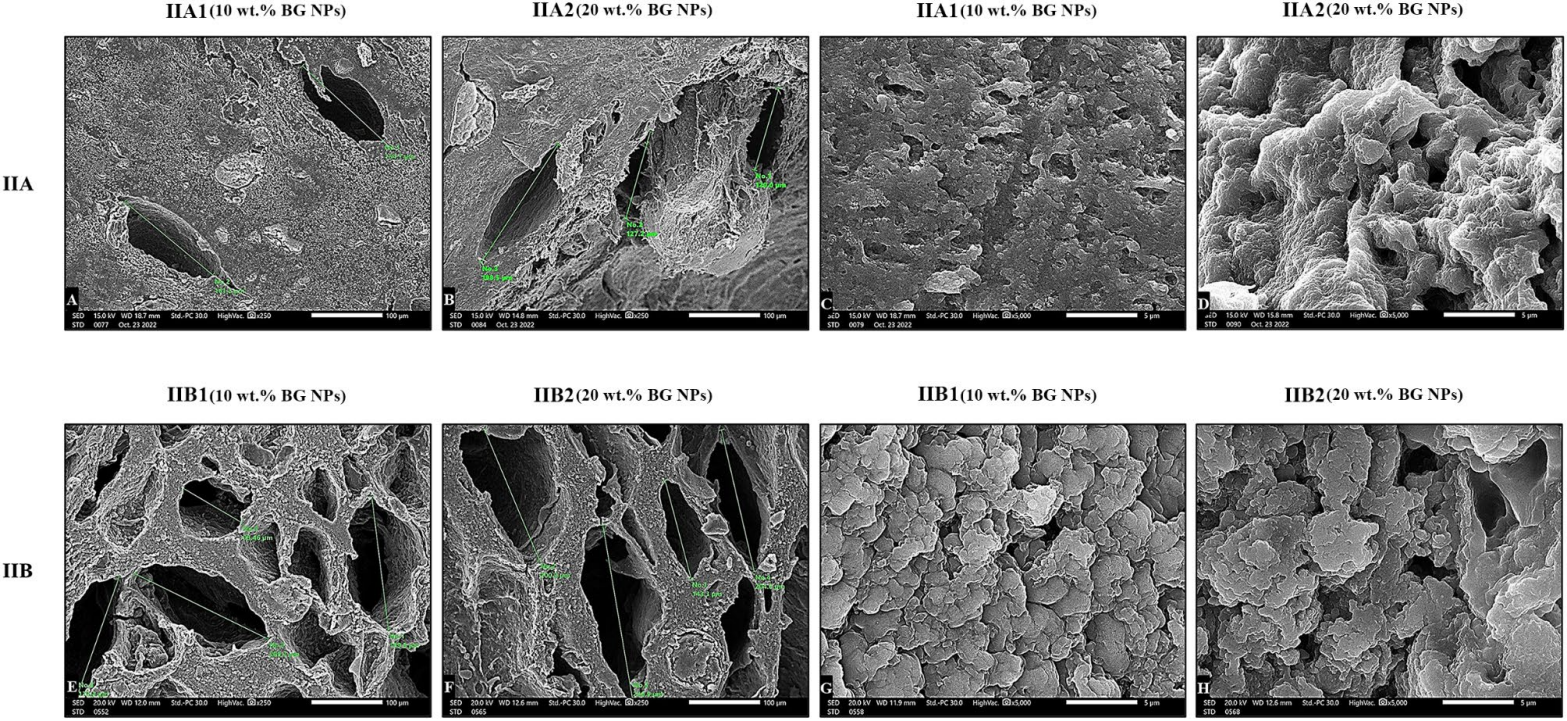
SEM images revealing surface morphology and pore size diameter of freeze-dried crosslinked sodium alginate hydrogel loaded with different MBG NPs, in various concentrations (10 and 20 wt%) as: Subgroup IIA1 [alginate/MBG NPs loaded hydrogel (10 wt%)] (**A** and **C)**; subgroup IIA2 [alginate/MBG NPs loaded hydrogel (20 wt%)] (**B** and **D**); subgroup IIB1 [alginate/BMBG NPs loaded hydrogel (10 wt%)] (**E** and **G**); subgroup IIB2 [alginate/BMBG NPs loaded hydrogel (20 wt%)] (**F** and **H**). **A**, **B**, **E**, **F** (cross-sectional view, original magnification X250); **C**, **D**, **G**, **H** (surface view, original magnification X5000)

Surface morphology and porosity of the constructed sodium alginate composite hydrogel are evaluated by SEM investigation. As shown in Fig. 3A and B, the porous structure and the decoration of BGNPs on alginate hydrogel surface are clearly observed. It can be clearly seen that BGNPs are adequately, homogenously dispersed in alginate matrix. SEM images of the unloaded sodium alginate hydrogel surface revealed a homogenous smooth surface, possessing multiple, micro-sized, circular to oval, interconnected pores with an average diameter of 4 μm (Fig. 4). All alginate/BGNPs loaded groups possess oval shaped macro–micro pores with well prominent walls and a large surface area as shown in (Fig. 5). It can be also observed that, the incorporation of MBG NPs has enlarged the pore size diameter [Subgroups: IIA1 (158 μm), IIA2 (149 μm)], compared to the control [Group I (4 μm)]. The increase in concentration of MBG NPs from 10 wt% to 20 wt% slightly decreases the pore size diameter from 158 μm, 149 μm to 152 μm, 147 μm (Fig. 5A-D). Also, a corrugated surface and a 3D porous structure with the interconnectivity of macro-micro pores are clearly seen (Fig. 5D). Alginate/BMBG NPS loaded subgroups (IIB1 and IIB2) exhibit the greatest increase in both porosity and pore size diameter (168, 183 μm; respectively), compared to all study groups [IIA1 (158 μm), IIA2 (149 μm), I (control) (4 μm)] (Fig. 5E-H) (Table 3). Moreover, it can be noted that in subgroups IIB1 and IIB2, pore size diameter increases from 168 μm (subgroup IIB1) to 183 μm (subgroup IIB2) as the concentration of BMBG NPs increase from 10 wt% to 20 wt% (Fig. 5E-H) (Table 3).

**Table 3:** Average pore size diameter of prepared composite hydrogels

Groups/subgroups		Average pore size diameter (µm) at magnification (X250)
NPs type/concentration (%)		
BG NPs	Concentration (%)	
Group I (Control)	0 wt% BG NPs	4
Group IIA1	10 wt% MBG NPs	158
Group IIA2	20 wt% MBG NPs	149
Group IIB1	10 wt% B-MBG NPs	168
Group IIB2	20 wt% B-MBG NPs	183

### Water uptake (%) of composite hydrogel

**Fig. 6:**
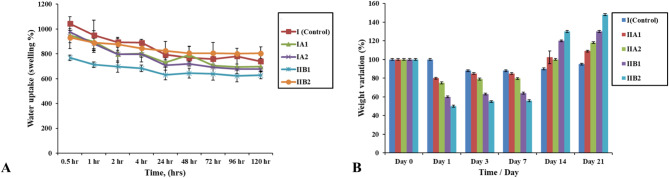
Water uptake (%) of all studied groups after incubation in distilled water at 37^o^C until an equilibrium swelling state was reached (**A**), weight variation percentage (%) of all studied groups after soaking in SBF solution for different time intervals (**B**)

Figure 6A presents the water uptake (WA %) results of the injectable sodium alginate composite hydrogel as a function of different MBG NPs being loaded in different concentrations (10 and 20 wt%). It can be clearly noted that all hydrogel specimens reach an equilibrium steady state after 120 h. The addition of BG NPs decreases the WA % of hydrogel except for subgroup IIB2 [alginate/BMBG NPs (20 wt%)], which shows the highest WA% (~ 800%) among all study groups/subgroups after 120 h. This sheds light on the role of boron in modulating the WA behavior (swelling) of the constructed sodium alginate composite hydrogel. It can be observed that, among group IIB, increasing concentration of the BMBG NPs greatly increased WA% [IIB1 (627.02), IIB2 (803.41) (WA %); respectively] but the opposite was demonstrated in group IIA [IIA1 (696.22), IIA2 (676.48) (WA %); respectively]. This is reinforced by the one-way *ANOVA* test displaying that the incorporation of BMBG NPs has significantly influenced the WA capacity of sodium alginate composite hydrogel (p value < 0.05).

### In vitro bioactivity and ions release analysis

#### Weight variation

Figure 6B reveals the weight variation of all hydrogel specimens after 0, 1, 3, 7, 14, and 21 days of immersion in SBF solution. It can be clearly displayed that all alginate/BGNPs loaded groups show a quick decrease in weight which is then maintained, followed by a notable increase up to 21 days relative to unloaded (control) group (0 wt% BG NPs). Alginate/BMBG NPs loaded hydrogels (group IIB), reveal the greatest and fastest weight loss after 1 and 3 days [IIB1 (40, 37) and IIB2 (50, 45) (%); respectively] followed by stabilization of weight then display the greatest increase after 14 and 21 days [IIB1 (20, 30) and IIB2 (30, 48) (%); respectively] with statistical significance (*p* < 0.05). Also results presented that increasing the concentration of BG NPs tend to influence weight as well, with subgroups, IIB2 and IIA2 exhibit the largest weight loss after 1, 3 days [IIB2 (50, 45) and IIA2 (25, 20.97) (%); respectively] with statistical significance (*p* < 0.05). Then stabilization of weight is supervened by the most pronounced weight gain after 21 days [IIB2 (48) and IIA2 (18) (%)]. This is approved by the *one-way ANOVA* test that has verified the significant effect (p value < 0.05) of BG NPs addition on the weight variation of hydrogel specimens after immersion in SBF with alginate/BMBG NPs loaded groups showing the most significant influence.

#### FTIR analysis

**Fig. 7:**
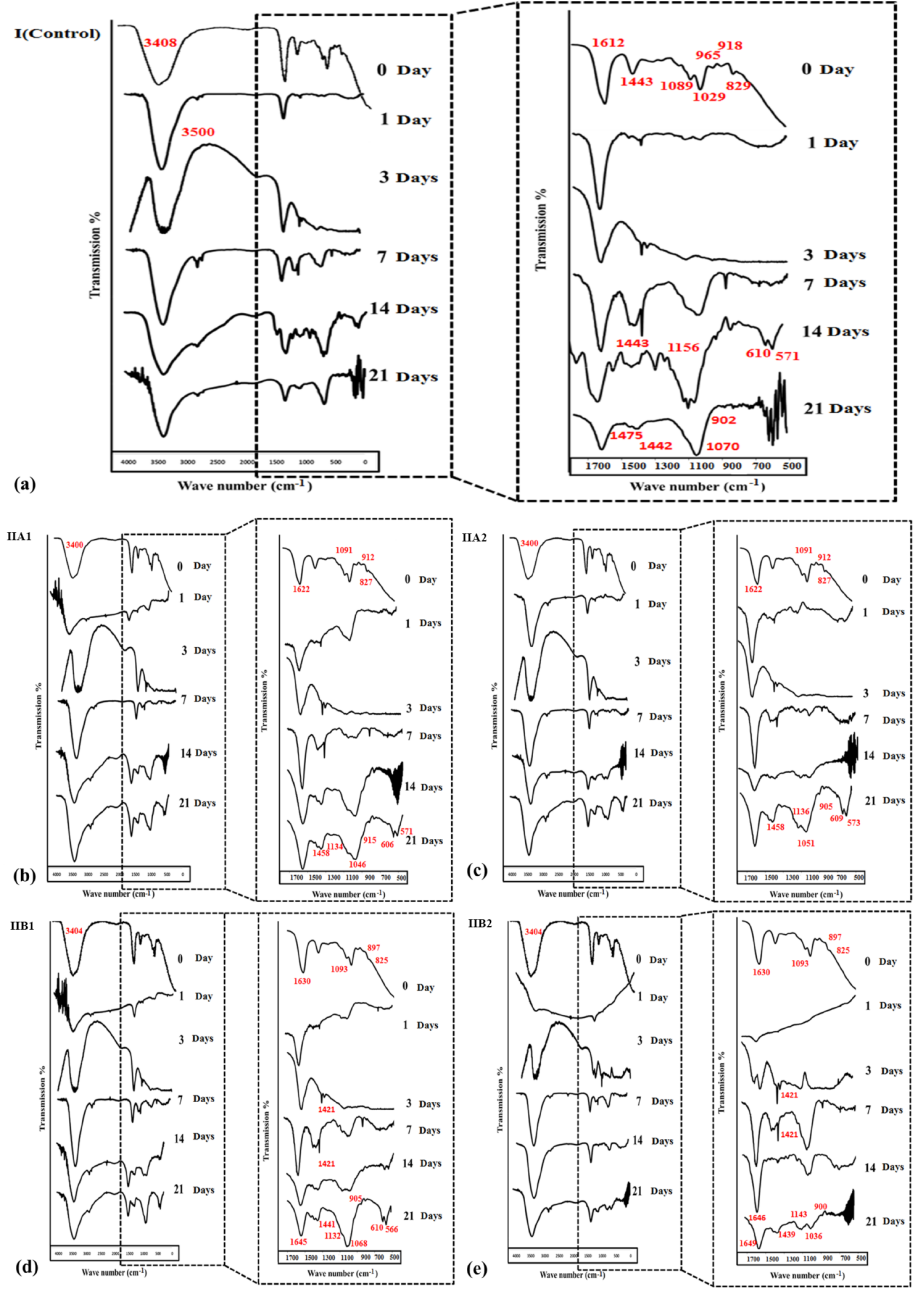
FTIR spectra of surfaces of specimens Group I: unloaded alginate hydrogel (**a**), Group IIA1: alginate/MBG NPs loaded hydrogel (10wt. %) (**b**), Group IIA2: alginate/MBG NPs loaded hydrogel (20wt. %) (**c**), Group IIB1: alginate/BMBG NPs loaded hydrogel (10wt. %) (**d**), and Group IIB2: alginate/BMBG NPs loaded hydrogel (20wt. %) (**e**) after incubation in SBF solution for times 0, 1, 3, 7, 14 and 21 days

Figure 7 shows the FTIR analysis of all hydrogel specimens before and after soaking in SBF for different time intervals. FTIR pattern of group I (Control) reveal an increase in ν 3500 cm^− 1^ band intensity after soaking in SBF for 1, 3, 7 days then a decline after 14 and 21 days (Fig. 7a). It can be clearly noted that the FTIR spectra of all alginate/BG NPs loaded groups/subgroups show the characteristic pattern of silicate-based bio glass with typical peaks at ν 1091–1093 cm^− 1^, ν 3400 cm^− 1^, ν 820 cm^− 1^, ν 897–912 cm^− 1^, and ν 1622–1630 cm^− 1^. These characteristic peaks (ν 1091–1093 cm^− 1^, ν 3400 cm^− 1^, ν 820 cm^− 1^, ν 897–912 cm^− 1^, and ν 1622–1630 cm^− 1^ ) correspond to Si-O stretching and overlapping of OH vibration from silanol and adsorbed water, Si-O bending, Si-O with one nonbridging oxygen, and molecular water (H-O-H scissoring); respectively (Fig. 7b-e). After soaking in SBF for 14 and 21 days, FTIR peaks have displayed changes in the hydrogel structure. All prepared hydrogels in all groups display transmission bands related to the vibrations of (PO_4_)^3−^ groups and (CO_3_)^2−^ groups which suggest the precipitation of carbonated apatite on top of hydrogels surface. Bands at ν 1458 –1439 cm^− 1^denote the stretching of C-O bonds (asymmetric stretching or v_3_) in (CO_3_)^2−^ group where bands at ν 900–915 cm^− 1^ denote the C-O bonds bending in (CO_3_)^2−^ group. After 14 and 21 days the intensities of bands ν 1132–1143 cm^− 1^ and ν 1036–1070 cm^− 1^, corresponding to asymmetric vibrational stretching or v_3_ of P-O bonds in (PO_4_)^3−^ group (phosphate precipitation) have been increased. In addition, double peaks exist at ν 571 cm^− 1^ and 610 cm^− 1^ (bending or v_4_) which represent the formation of HA precipitates. Borate doped MBGNPs loaded groups show a decline in stretching vibrations of B-O bonds in [BO_3_] units ν (1439–1441 cm^− 1^) that correspond to the fast hydrolysis of the hydrogel network which is in harmony with the quick release of boron (Fig. 7d and e).

#### SEM-EDX analysis

**Fig. 8:**
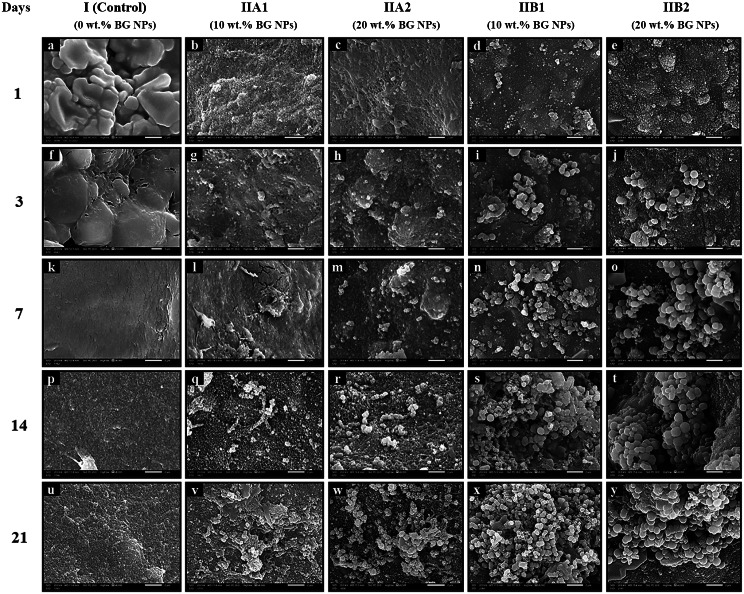
SEM images (surface view) of freeze-dried specimens of crosslinked unloaded alginate hydrogel (control) and alginate composite hydrogel loaded with different types and concentrations of MBG NPs after immersion in SBF for times 1, 3, 7, 14 and 21 days, as unloaded sodium alginate hydrogel (Group I) (control) (**a**,** k**,** p**,** u** original magnification X8000 and **f** original magnification X10000); alginate/MBG NPs loaded hydrogel (10 wt%) (Subgroup IIA1) (**g**,** l**,** q**,** v** original magnification 8000 and **b** original magnification X9500); alginate/MBG NPs loaded hydrogel (20 wt%) (Subgroup IIA2) (**c**,** h**,** m**,** r**,** w** original magnification X8000); alginate/ BMBG NPs loaded hydrogel (10 wt%) (Subgroup IIB1) (**d**,** i**,** n**,** s**,** x** original magnification X8000**)**; alginate/BMBG NPs loaded hydrogel (20 wt%) (Subgroup IIB2) (**e**,** j**,** o**,** t**,** y** original magnification X8000)

SEM images present the surface morphology of the constructed hydrogel surfaces after immersion in SBF for 1, 3, 7, 14 and 21 days (Fig. 8). It can be noted that the surface morphology has been altered with the appearance of globular structures of Ca/P layer on the hydrogel surface. These precipitates become thicker and homogenously distributed with increasing BG NPs concentration and soaking time. It can be obviously revealed that the unloaded alginate hydrogel surface shows small, poorly remarkable, less evident areas of Ca/P deposits after only 14 and 21 days, compared to all other test groups (Fig. 8p and u). It can be apparently noticed that all alginate/BMBG NPs loaded groups (IIB1 and IIB2) have started to display the earliest obvious Ca/P crystal growth, only after 3 days of soaking in SBF (Fig. 8i and j). But for other alginate/MBG NPs loaded groups (IIA1 and IIA2) crystals begin to appear after 7 days (Fig. 8l and m). In addition, groups (IIB1 and IIB2) convey the largest, most dense, well prominent clusters of spherical hydroxyapatites (HA) particles (grape-like structure) after 21 days. These HA clusters (grape-like structure) cover the hydrogel surface fully and homogenously (Fig. 8x and y). It can be apparently seen that after 14 days subgroup IIB1 exhibits (*pseudo*-spherical) and (*cauliflower*-like) aggregates of hydroxycarbonate apatite (HCA) crystallites which are enlarged after 21 days (Fig. 8s and x). This has happened because of more nucleation of calcium phosphate forming aggregates of large spherical HA particles. Subgroup IIA2 shows, unlike alginate/BMBG NPs loaded groups, small *cauliflower*-like (*lath*-like) crystals, typical structure for apatite–like layer, that have covered small regions of the surface after 14 days (Fig. 8r). After 21 days most of the hydrogel surface in subgroup IIA2 has been covered with the small cauliflower-like crystals (Fig. 8w).

Figures S3-S6 (supplementary data) display the results of the elemental composition of the precipitated apatite layer on top of all prepared hydrogel discs after 14 and 21 days of immersion in SBF. It is clear that calcium, phosphate and oxygen are the major ions that are recognized in the precipitated layer attributed to calcium phosphate. In Fig. S7 (supplementary data), it can be obviously noted that the Ca/P ratios of the precipitates being detected on group I (Control) and subgroup IIA1 both after 14 and 21 (days) are (1.33, 1.35 and 1.4, 1.45; respectively). These ratios (1.33, 1.35 and 1.4, 1.45; respectively) seem less than Ca/P ratio for stoichiometric hydroxyapatite (1.67). Ca/P ratio of the precipitates being found on subgroups IIA2 (on day 21) and IIB1 (on day 14) (1.6) are like Ca/P ratio for the stoichiometric pure hydroxyapatite (1.67). Ca/P ratios of the precipitates being formed on the other groups are in the range of 1.0 to 3.0, that are distinctive to that of HCA composition of human bone (1.5) and other phases of calcium phosphates present in the body. The one-way *ANOVA* assist that both incorporating and increasing BG NPs concentration have significantly increased bioactivity of the sodium alginate hydrogel with boron doped MBG NPs conveying the most favorable impact on bioactivity (p value < 0.05).

### In vitro ion release and pH evaluation

**Fig. 9:**
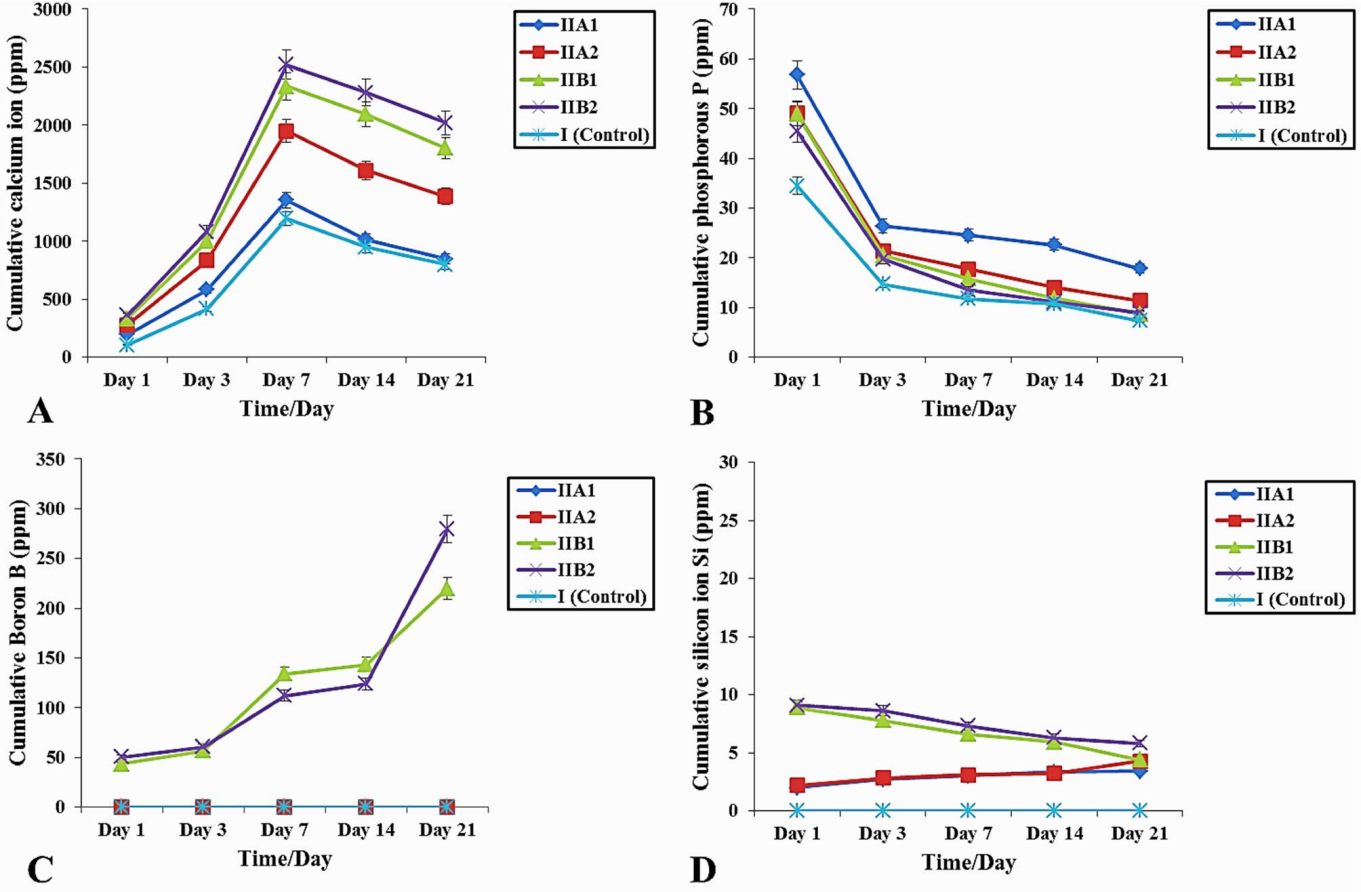
Temporal ions released calcium (**A**), phosphorous (**B**), silicon (**C**), and boron (**D**) concentrations (ppm) in SBF after immersion of prepared hydrogel specimens of all study groups

Specimens are immersed in SBF for different time intervals (1, 3, 7, 14 and 21 days), filtered and solute examined by inductively coupled plasma-mass spectroscopy (ICP-MS). In Fig. 9A, the release of calcium ion shows the classic behavior for bioactive glasses. It can be clearly seen that the release of calcium ion from all alginate/BG NPs loaded composite hydrogel groups increase till day 7, then greatly decrease till day 21. Boron plays a major role in the release/re-uptake mechanism of calcium ion (profile). Group IIB exhibits the best release/re-uptake calcium ion profile, compared to all other studied groups. Figure 9B presents the phosphate ion release profile. It can be clearly noted that after 21 days, subgroups IIB2 and IIA1 represent the least and greatest phosphate ion concentration in SBF 8.95 and 17.82 (mg/L); respectively. Results also note that after immersion in SBF for 21 days subgroup IIB2 has manifested greater phosphate ion concentration (8.95 mg/L), in comparison to subgroup IIB1 (8.91 mg/L). Figure 9C displays the boron ion release profile, the release of boron ion constantly and steadily increases as soaking time in SBF increases. Subgroup IIB2 achieves the superior boron ion release after 21 days (279.51 mg/L) relative to subgroup IIB1 (219.8 mg/L). Figure 9D reveals silicon ion release behavior. As shown, a slight increase in silicon ion concentration is observed till days 7, then being nearly sustained. This could suggest that part of silicon ions has become obscured from dissolution by the newly precipitated HA layer on the surface of specimen. Like, calcium ion release pattern, boron plays an important role in silicon ion release mechanism. After 21 days it is obvious that the greatest and least silicon ion release is found in subgroups IIB2 (5.81 mg/L) and IIA1 (3.42 mg/L); respectively. Also, it can be clearly shown that in group IIB silicon ion release increased from 4.41 mg/L (subgroup IIB1) to 5.81 mg/L (subgroup IIB2) as the concentration of BMBG NPs nanoparticles increases from 10 to 20 wt%.

### pH evaluation

Figure S8 (Supplementary data) presents SBF pH results after immersion of hydrogel specimens for 1, 3, 7, 14, and 21 days. Initially the pH of the SBF is 7.4 then it increases for all loaded hydrogel specimens, compared to control group up to 21 days. The pH of boron doped BG NPs loaded groups is slightly lower than other groups specially after 7 and 14 days of immersion in SBF with statistical significance (*p* < 0.05). The change in pH remained, for all groups/subgroups, within the range of 7.0 and 8.0.

### Cell viability (%) by MTT assay of composite hydrogels

**Fig. 10:**
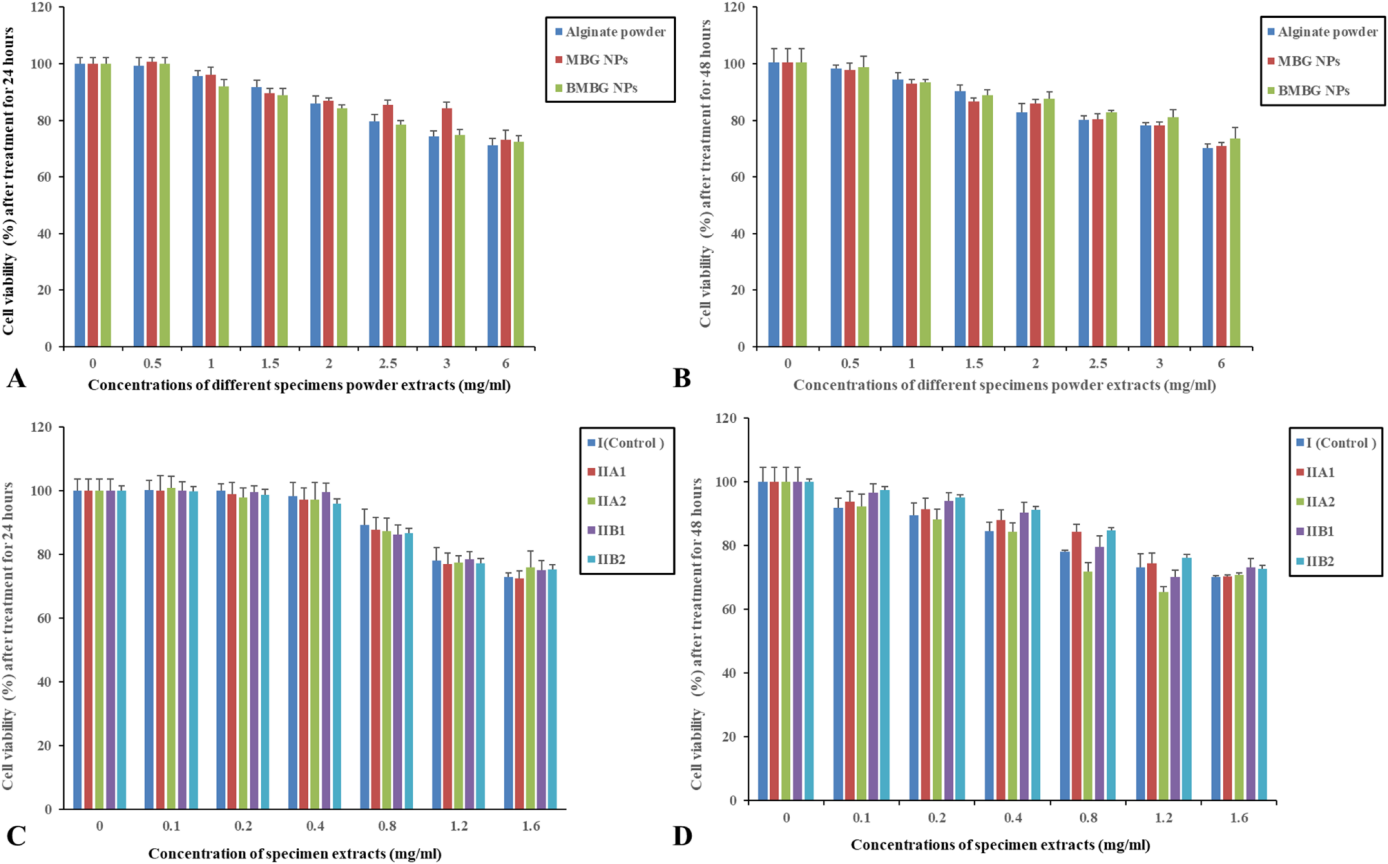
Effect of specimen powder extracts on the viability of gingival cells (**A**,** B**); effect of prepared specimen extracts on the gingival fibroblast cells (**C**,** D**). Cells were exposed to the tested specimens at different concentrations (mg/mL) for 24 and 48 h. The cell viability test was assessed by using MTT assay. All values represent the average values from 3 experiments and expressed as mean ± SEM

Cell viability % of human gingival fibroblast cells after 1 and 2 days of incubation in different concentrations of sodium alginate powder, MBG NPs, BMBG NPs and composite hydrogels extracts from all groups/subgroups are shown in Fig. 10. The optical density values are derived from the MTT assay of cells incubated in the extracts of powders and hydrogels from all study groups/subgroups. It can be clearly observed from MTT assay data that the cell viability % of human gingival fibroblast cells have declined with increasing the concentration of both powders and hydrogel extracts in all the groups/subgroups after 1 and 2 days, but still higher than the accepted cell viability % of (> 70%), according to (ISO 10993-5(E)). This is supported by the one-way *ANOVA* test emphasizing that incorporation of all MBG NPs in different concentrations (10 wt%, 20 wt%) has affected cell viability of human gingival fibroblast cells positively. Outcomes were statistically significant (p value < 0.05).

## Discussion

In the present study, we were able to formulate, construct and characterize, adequately, a novel injectable sodium alginate hydrogel loaded with different concentrations (10 wt% and 20 wt%) of both NT-MBG 58S3.6 (MBG NPs) and NT-MBG 44S3.6 B14 (B-MBG NPs) nanoparticles as a pulpotomy filling material for dentin regeneration.

Sodium alginate (SA) was chosen in the current work because it has got many advantages that advocate its use as a scaffold in the bio-regenerative fields [7]. However, it displayed some disadvantages such as inferior mechanical properties [27], low porosity, small pore size diameter [28] and retarded degradation [29]. Substantially, to overcome these defects sodium alginate was loaded with various % of MBG NPs as inorganic nanofillers [30].

Furthermore, boron doped-mesoporous bioactive glass NPs (NT-MBG 44S3.6 B14) were selected, due to their great eminent biological effect in increasing the release of growth factors and cytokines, stimulating bone regeneration. This biological effect was evidenced by Dzondo-Gadet and co-workers [31], who recognized the liberation of growth factors VEGF and TGF-ß after the use of boric acid that were useful for angiogenesis and wound healing [14, 31].

In the present study an optimum condition of (7 wt% sodium alginate: 20 wt% calcium chloride dihydrate) was selected which facilitated the achievement of our novel injectable in situ developing hydrogel that precisely impregnated all structural unreliable details in the operative field. Furthermore, they assured that all predecessors were sufficiently blended and hydrogel characteristics were not negatively affected.

The technique applied here was like that followed by Fu et al. [16] and Sánchez-Fernández J.A et al. [15], which ensured homogenous distribution of BG NPs inside the hydrogel matrix. A specially constructed sterile dual–syringe system was utilized, like that used by Cardoso DA et al. [17], but with some modifications which resulted in a smooth, continues, easy and efficient ejection of a homogenous, consistent formulation. Furthermore, it ensured that an adequate in situ crosslinking of sodium alginate phase had been established. Moreover, it ascertained efficient delivery of the hydrogel to target areas, hence making it more clinically applicable [32].

According to FTIR analysis, characteristic bands of sodium alginate [33] and all added BG nanoparticles [34] were present in the FTIR spectrum of the newly constructed composite scaffold (Fig. 2). This approved the collaboration between sodium alginate matrix and BG NPs being added. Notable physicochemical changes were recognized following crosslinking of prepared specimens in calcium chloride solution that committed the crosslinking of alginate polymer chains [35]. There was a slight transfer in most of transmittance peaks in the crosslinked specimens. This movement was related to the movement of carboxyl group [36]. Consequently, this cross linking made the scaffolds insoluble [37] and converted them into stable hydrogels [35].

Additionally, the appearance of the two peaks at ν 1431 cm^− 1^ and 1600 cm^− 1^ indicated the asymmetric and symmetric stretch vibration of –COO^−^ related to carboxylic acid salts [38], that are distinct to ionic binding [39]. Also, the peak at 1079 cm^− 1^ was attributed to C-C and C-O stretching which emphasized the crosslinking between sodium alginate and calcium chloride. Furthermore, the peak of C-C stretching at ν 1022 cm^− 1^ submitted a powerful bond between Ca^+ 2^ and guluronic acids of sodium alginate [40]. In addition, the band at ν 3256 cm^− 1^ of (O-H stretching) was shifted to ν 3344 cm^− 1^ that corresponded to the declined intramolecular bonding [41]. Current results were consistent with alginate/BG composites (organic-inorganic beads) prepared by Costa et al. [42], showing similar characteristic IR peaks.

SEM analysis revealed that we succeeded in overcoming the nano-size pore obstacle in sodium alginate-based hydrogels [28] (Fig. 4). This was affirmed by the remarkable increase in pore size diameter in all alginate/BG NPs loaded hydrogel groups/subgroups (Fig. 5) (Table 3). Moreover, all BG NPs loaded hydrogel groups possessed oval shape micro–macro pore configuration with well prominent walls and a large surface area. This obtained configuration is needed for migration and organization of dental stem cells, neurogenesis, angiogenesis and diffusion of nutrients throughout the scaffold structure [43]. Furthermore, the homogenous hydrogel surface could suggest the optimal compatibility between the incorporated BG NPs and the alginate matrix [42].

Boron demonstrated a major role as it was clear that BMBG NPs loaded group (IIB) displayed the most apparent increase in both porosity and pore size diameter (Fig. 5E-H) (Table 3) among all groups. Also, it can be evidently noted that pore size diameter was increased from 168 μm in subgroup IIB1 to 183 μm in subgroup IIB2 as the concentration of BMBGNPs was raised from 10 wt% to 20 wt%. Similarly, alginate/MBG NPs loaded hydrogel group (IIA) demonstrated an enhanced porosity. Nevertheless, a decline in pore size diameter was observed from 158 μm in subgroup IIA1 to 149 μm in subgroup IIA2 when the concentration of MBG NPs increased from 10 wt% to 20 wt% (Fig. 5A-D) (Table 3). Our results were consistent with Tian T et al. [44] who discovered that the incorporation of micro-nano bioactive glass (MN BG) to poly (lactic-co-glycolic acid) (PLGA) scaffold significantly enlarged pore size diameter. This finding was attributed to the diminished interlaced chains of PLGA scaffold due to MN BG which restricted their motion, bringing about a big ice crystal and hence a great pore size diameter [44]. On the other hand, these outcomes were averse to Lemos and colleagues [45], who found that porosity of the constructed 3D nanocomposite chitosan/bioactive glass scaffolds was reduced with the addition of BG NPs. They attributed this outcome to the increased density of the scaffolds after bioactive glass inclusion [45, 46]. Consequently, we managed to achieve the desired porosity and pore size diameter which exceeded the least pore size (50 μm) needed to permit the infiltration and growth of mineralized tissues [47].

Water uptake capacity was carefully investigated as an essential characteristic that had a strong control over hydrogel performance with a great impact reflected on their mechanical properties [48]. In fact, hydrogels with their 3D and hydrophilic nature were capable of absorbing and keeping an adequate quantity of water inside. This brought about a great water uptake percentage which helped in nutrient transfer [49]. As well, it increased pore size that tended to elevate interior surface area essential for cell infiltration and adaptation [50]. It was clearly noticed from the current results that the addition of BG NPs and the increase in their concentration, except for alginate/BMBG NPs loaded group (20 wt%) had decreased the WA%, compared to unloaded hydrogel which was statistically significant (*p* < 0.05) (Fig. 6A). This could be explained by the reduced polymer surface needed for joining water molecules [51]. Another explanation could be related to the liberation of calcium ions during the dissolution of BG NPs which acted as a donor ion provider (reservoir of ions) needed for further hydrogel crosslinking. This calcium ions liberation had a negative effect on the usual osmotic swelling behavior of alginate supported hydrogels [52]. On the contrary, alginate/BMBG NPs loaded groups showed a remarkable increase in WA% as the concentration of NPs was elevated from 10 wt% to 20 wt% which was statistically significant (*p* < 0.05). This was in consonance with much published research; Ghimire [53], and Hafezi et al., [54] displayed that water absorption capacity of composite scaffolds was improved after the addition of greater BG proportions. Also, Moonesi Rad et al. [14], conveyed an obvious elevation in WA% with increased concentration of borate modified BG NPs from 10 wt% to 20 wt%. First, they all contributed this to the BG hydrophilic characteristic. Second, to the wide BG/polymer link and the fragile mechanical interconnection among BG and polymer matrix. Beside all above mentioned reasons, the distinct increase in porosity and pore size diameter that was concealed in the current SEM images (Fig. 5A-H) (Table 3) of alginate/B-MBG NPs loaded groups, discussed before, could be an important cause which allowed additional water impregnation that further enhanced the swelling of the hydrogel [48], and created enough room for odontoblast arrangement in dentin regeneration [55].

In the current work SBF soaking test was used to assess in vitro bioactive potential of the constructed hydrogel [22, 56], based on: weight variation measurement, FTIR analysis, apatite layer precipitation on top of material, calcium to phosphorous ratio (Ca/P) determination, ion release assessment and pH evaluation.

For weight variation measurement, the recognizable initial reduction in weight presented in all alginate/BG NPs loaded groups could be ascribed to the BG dissolution and ion release in SBF. Then the stabilization of weight could be referred to the balance between salt release and the precipitation of HA coat on top of hydrogel. After 14 days, weight was promptly elevated, in all alginate/BG NPs loaded groups, attaining around 50% weight gain with statistical significance (*p* < 0.05). This could be contributed to the strong bioactivity of BG NPs that transferred the initial loss followed by the equilibrium in weight of the hydrogel specimens towards the great increase in weight. This remarkable weight increase corresponded to the precipitation of hydroxyapatite on top of the modified hydrogel surface, preferred by interchange of SBF which enhanced the mechanics of HA deposition [57] (Fig. 6B). Current outcomes were in line with Furlan et al. [57], who conveyed great in vitro bioactive potential of sol-gel prepared boron-based 45S5 bio-glass (45S5B).

Boron played an important role concerning weight variation with alginate/BMBG NPs loaded groups revealing the greatest and fastest weight loss after 1, 3 days [IIB1 (40,37) and IIB2 (50,45) (%); respectively] with statistical significance (*p* < 0.05). This could be brought about probably, due to frail glass morphology. This frail construction could have been resulted from B_2_O_3_ replacement (small proportion of SiO_2_) [58] and the ability of boron to alter the glass system arrangement, binding –OH groups to the glass surface [12]. All these might tend to induce the glass bioactive potential [59]. Additionally, more terminal groups (-OH) could be found and not fully removed during synthesis of BG NPs, which were more liable to react with SBF solution [60]. Then stabilization of weight was displayed. Finally, the greatest increase in weight was administered after 14 and 21 days [IIB1 (20,30) and IIB2 (30,48) (%); respectively] that was related to the great deposition of hydroxyapatite (HA) precipitates, and this was statistically significant (*p* < 0.05). These outcomes were in line with various studies who exhibited that boron doped BG degraded faster than silicate glasses and fully transformed into HA [61, 62]. They related this to the unstable combination of trigonal planar [BO_3_] and tetrahedral [BO_4_] units within BBG which decreased network interconnectivity [61, 63].

Chemical analysis (FTIR) is one of the most accurate methods to detect the formed carbonated hydroxy calcium phosphate precipitate all through the whole surface of hydrogel specimen [62]. FTIR spectra of all alginate/BG NPs loaded hydrogel groups showed characteristic bands attributed to both phosphates bending vibration and carbonate group (Fig. 7). This indicated the development of carbonated hydroxyl calcium phosphate deposits on top of alginate/BG NPs loaded hydrogels which were markedly noticed among borate doped MBG NPs loaded groups especially with higher BMBG NPs content (20 wt%) (Fig. 7d and e). This was in harmony with Yu et al. [64], who reported that bands set near to ν 562 cm^− 1^, 602 cm^− 1^, and 1044 cm^− 1^ were representative of a strong crystalline hydroxyapatite. Noteworthy, there was a remarkable increase in the ν 3500 cm^− 1^ band intensity, in FTIR pattern of unloaded alginate (control group), after soaking in SBF for 1, 3, 7 days, followed by a decrease after 21 days. This could be related to the degradation of the inceptive portion of alginate matrix in the first 7 days specifically the part with low crosslinking degree. Then after 21 days of immersion in SBF the remaining part of alginate with the greatest gelation degree and least carboxylate groups existed that restricted the polymer chains to confine water molecules [64] (Fig. 7a).

A combination of microscopic examination and chemical (structural) analysis (SEM-EDX) precisely assessed the formed carbonated hydroxy calcium phosphate precipitate at the surface of hydrogel. It provided the reader with a clear picture and elemental composition (Ca/P ratio) of the precipitated apatite layer [65].

SEM examination results, of the current in-vitro bioactivity test, evidenced the slight bioactive capability of sodium alginate hydrogel. This was indicated by the less apparent areas of Ca/P deposits noticed on the unloaded alginate hydrogel surface (control) after 14–21 days relative to all alginate/BG NPs loaded hydrogel specimens (Fig. 8p and u). This demonstrated the capacity of sodium alginate, solely, to initiate nucleation like crystal seed. A clear enhancement in bioactive potential of alginate hydrogel after the addition of BG NPs was indicated by the formation of globular structures of Ca/P layer on the hydrogel surface which became thicker and homogenously distributed with increasing BG NPs concentration and soaking time.

Alginate/BMBG NPs loaded groups exhibited the strongest bioactive capability among all BG NPs loaded groups with subgroup IIB2 (BMBG NPs 20 wt%) displayed the best bioactivity. This was affirmed first by the earliest obvious Ca/P crystal growth seen in subgroup IIB2, after 3 days of immersion in SBF compared to other alginate/MBG NPs loaded groups (Fig. 8j). Second, prominent clusters of spherical HA particles (*grape-like* structure) covered fully and homogenously hydrogel surfaces of subgroup IIB2 after 21 days (Fig. 8y). This could be explained by the declined proportion of SiO_2_ derived from B_2_O_3_ substitution which brought about frail glass interconnectivity [66]. In other words, the existence of boron in the BG structure might have altered the structural arrangement and joined hydroxyl groups to BGs surface [12]. This agreed with Gu Y et al. [67], who revealed that 13-93B3 fibers were completely transferred to HA, oppositely 13–93 fibers were partly transformed.

EDX elemental analysis supported the visual image displayed by SEM examination. It was clear that calcium, phosphate and oxygen were the major ions that were recognized in the precipitated layer which was attributed to calcium phosphate. The unloaded sodium alginate hydrogel exhibited Ca/P ratio after 14–21 days (1.33 and 1.35; respectively) that was so far from the stoichiometric hydroxyapatite (1.67) [68], with statistical significance (*p* < 0.05). This could affirm sodium alginate hydrogel weak bioactivity. Favorably, all alginate/BG NPs loaded groups that showed great enhancement of bioactivity with great, notable mineral deposits in SEM examination, conveyed a Ca/P ratio of (1.4–3.0). This obtained Ca/P ratio (1.4–3.0) was very close to that of hydroxycarbonate apatite (HCA) (1.5) of human bone [69], and different phases of calcium phosphates present in the body [70], with a statistically significance (*p* < 0.05). It is worth mentioning that Ca/P ratio of the precipitates found on subgroups IIB1 (on day 14) and IIA2 (on day 21) (1.6) were close to Ca/P ratio for the stoichiometric pure hydroxyapatite (1.67). Also, the promising effect of increasing BG NPs content on bioactivity was approved by the increase in the Ca/P ratio which was statistically significant (*p* < 0.05). Our findings were in accordance with Moonesi Rad et al. [14], who reported that BG treated groups displayed the best bioactive potential and (10–20) % borate alteration had a favorable influence on the bioactivity.

Generally, the time- related development of calcium, phosphate and silicon ion concentrations (Fig. 9A, B, D) displayed the common configuration for BGs [23]. Calcium ions liberated from all study groups were high for the period 1–7 days of immersion which indicated the great calcium ion release from both BG NPs dissolution and sodium alginate hydrogel. This increase was positively correlated to the BG NPs content in the hydrogel (Fig. 9A).

Similarly, boron ions followed the same trend (Fig. 9C). Then the significant decline in both calcium and phosphate ions liberation can be clarified as the consumption of both ions in the formation and precipitation of calcium-phosphates (such as hydroxycarbonate apatite, HCA) (Fig. 9A and B). Boron as well as BMBG NPs content had a major role in the release/re-uptake mechanism of calcium and phosphate ions as shown that subgroup IIB2 displayed the highest ion release/ re-uptake configuration. Different reasons could be responsible for this discharge behavior: (1) lack of boron contribution in silica gel layer development which resulted in a decline in the thickness of the Si-O-Si gel layer. This gel layer was considered as a boundary that prevented ions exchange; (2) the slacked BG morphology resulted from the development of [BO_3_] unites; (3) a reduction in the chemical durability of BGs caused by B_2_O_3_ replacement [60]; (4) the reaction energy barrier that declined in the order Si-O-Si > Si-O-B > B-O-B [71]; (5) the decline in the connection probability of [SiO_4_]–[SiO_4_] and [SiO_4_]-[BO_x_] that followed the increase in B_2_O_3_ amount [72]; (6) the elevated B_2_O_3_ resulted in a low diffusion energy barrier for glass former ions which was associated as well with their high diffusion coefficient [72]; and (7) the absence of ring morphology in the BMBG NPs construction after the increase in the NPs content [72].

Our findings were consonant with Sakthi Prasad S et al. [73], who observed a regular elevation in ion content (such as Ca^2+^, B^3+^) in SBF solution associated with a regular elevation in B_2_O_3_ replacement [14, 73]. Conversely, Deilmann et al. [23] indicated that large boron content restricted the Ca ion reuptake and the consequent development of HCA layer.

Silicon ion was regarded as a powerful measure of BG chains dissolution [14]. The slight increase in silicon ion concentration which was then nearly sustained could be either contributed to two reasons. First, this might be due to the deposition of insolvable salts containing Si. Second, this might be related to the newly precipitated HA layer on the surface of specimen which obscured part of Si ions from dissolution [74]. Like, calcium, phosphate and boron ions, silicon ion discharge behavior was dependent on both boron and B BG NPs concentration in the hydrogel [14] (Fig. 9D).

Obvious increase in pH of the SBF solution occurred over time after the immersion of all hydrogel specimens from all study groups, which was statistically significant (*p* < 0.05) on days 7 and 14 [Fig. S8 (Supplementary data)]. This finding could be related to the ion interchange among hydrogen in SBF solution and BG exterior and the in vitro development of HA deposits on top of hydrogel surface [75]. Additionally, sodium and calcium ions interchange with hydrogen ions at the early disintegration period might have caused a rise in pH [76]. While the slight drop in pH in borate doped BG NPs treated groups that was presented on day 3 could be assigned to the breakdown of boron oxide leading to formation of boron acid by-products (e.g. H_3_BO_3_), this was not statistically significant [77]. Worthwhile, the precipitated salts were able to conserve the pH, for all study groups, within the limit of 7.0 and 8.0 which was appropriate for cell proliferation and differentiation [57].

Eventually, results of weight variation, FTIR, SEM/EDX, ion discharge and SBF pH measurements affirmed one another. As calcium ion content, in SBF, started to decrease, phosphate bending vibrations and carbonate groups were revealed in the FTIR spectra. Also, Ca/P deposits which were visible in SEM images, were also confirmed by characteristic peaks of calcium and phosphate in EDX spectra in similar time intervals. These were accompanied by an increase in SBF pH value.

Biocompatibility is an essential requirement in tissue engineering and various studies aimed to construct injectable hydrogels from biocompatible materials [78]. Based on MTT cell viability assay results (Fig. 10), the newly constructed injectable alginate hydrogel loaded with MBG NPs and BMBG NPs in different concentrations (10 and 20 wt%) proved to be highly biocompatible with statistical significance (*p* < 0.05). Although increasing the concentration of both nanoparticles and hydrogel extracts in all the groups/subgroups after 1 and 2 days shows a decline in the cell viability % of human gingival fibroblast cells but kept (persisted) higher than the accepted cell viability % of (˃ 70%) according to (ISO 10993-5(E)).

## Conclusions

The pivotal effect of boron, in the regenerative field, was affirmed by the promising laboratory performance of the novel injectable alginate composite hydrogel. Alginate/BMBG NPs (20 wt%) (IIB2) showed a non-cytotoxic effect on gingival fibroblast cells (˃70%) according to (*ISO 10993-5(E)*). Additionally, this group (IIB2) exhibited the strongest bioactive potential displaying the earliest obvious Ca/P crystal growth, as observed in SEM micrographs. Also, prominent clusters of spherical HA particles fully and homogenously covered hydrogel surfaces after 21 days. Moreover, Ca/P ratio of precipitates in subgroups IIA2 (on day 21) and IIB1 (on day 14) (1.6) were like Ca/P ratio for stoichiometric pure hydroxyapatite (1.67). BMBG NPs incorporation greatly increased pore size diameter and WA% of constructed alginate hydrogel which are crucial requirements in scaffold fabrication for tissue regeneration purposes. The current search for a new biomaterial in minimally invasive regenerative *pulpotmy* (vital pulp therapy) is the prime aim of various research. Further in vivo animal studies are required, as an appropriate transitional step prior to clinical trials, for assessing the capability of the current novel formulated scaffold, alginate/BMBG NPs (20 wt%), for dentin regeneration. As their outcomes could be more reliably utilized in clinical implementation.

## Electronic supplementary material

Below is the link to the electronic supplementary material.

Supplementary Material 1

## Data Availability

The datasets used and/or analyzed during the current study are available from the corresponding authors (E.A. Kamoun and Mona Mohy El-Din) on reasonable request.

## Abbreviations

MBG NPs: Mesoporous bioactive glass nanoparticles
BMBG NPs: Boron doped-mesoporous bioactive glass nanoparticles
WA%: Water uptake percentage
BG NPs: Bioactive glass nanoparticles
MTA: Mineral trioxide aggregate
SBF: Simulated body fluid
TRIS: Tris–Hydroxymethyl aminomethane
MTT: (3-(4,5-dimethylthiazol-2-yl)-2,5-diphenyltetrazoliumbromide)
DEMSO: Dimethylsulfoxide
FTIR: Fourier transform infrared spectroscopy
SEM: Scanning electron microscope
EDX: Energy dispersive X-ray spectroscopy
ICP-MS: Inductively coupled plasma-mass spectroscopy
APHA: American Public Health Association
ELISA: Enzyme linked immunosorbent assay
SD: Standard deviations
HCA: Hydroxycarbonate apatite
