# Supplementary material for: A novel injectable boron doped-mesoporous nano bioactive glass loaded-alginate composite hydrogel as a pulpotomy filling biomaterial for dentin regeneration
Source: BMC Oral Health. 2024 Sep 14;24:1087. doi: 10.1186/s12903-024-04808-3 (PMC11401322; doi:10.1186/s12903-024-04808-3)
Supplement: Supplementary file 1 — Supplementary Material 1 [file 12903_2024_4808_MOESM1_ESM.docx]

**A novel injectable boron doped-mesoporous nano bioactive glass loaded-alginate composite hydrogel as a pulpotomy filling biomaterial for dentin regeneration: Formulation, characterization and *in vitro* performance of Part I (Laboratory study)**

Marwa S. Naga^1*^, Hala M. Helal^2^, Elbadawy A. Kamoun^3,4*^, Maha Abdel Moaty^1^, Samia S. Abdel Rehim Omar^5^, Ahmed Z. Ghareeb^6^, Esmail M. El-Fakharany^7,8^, Mona Mohy El Din^1*^


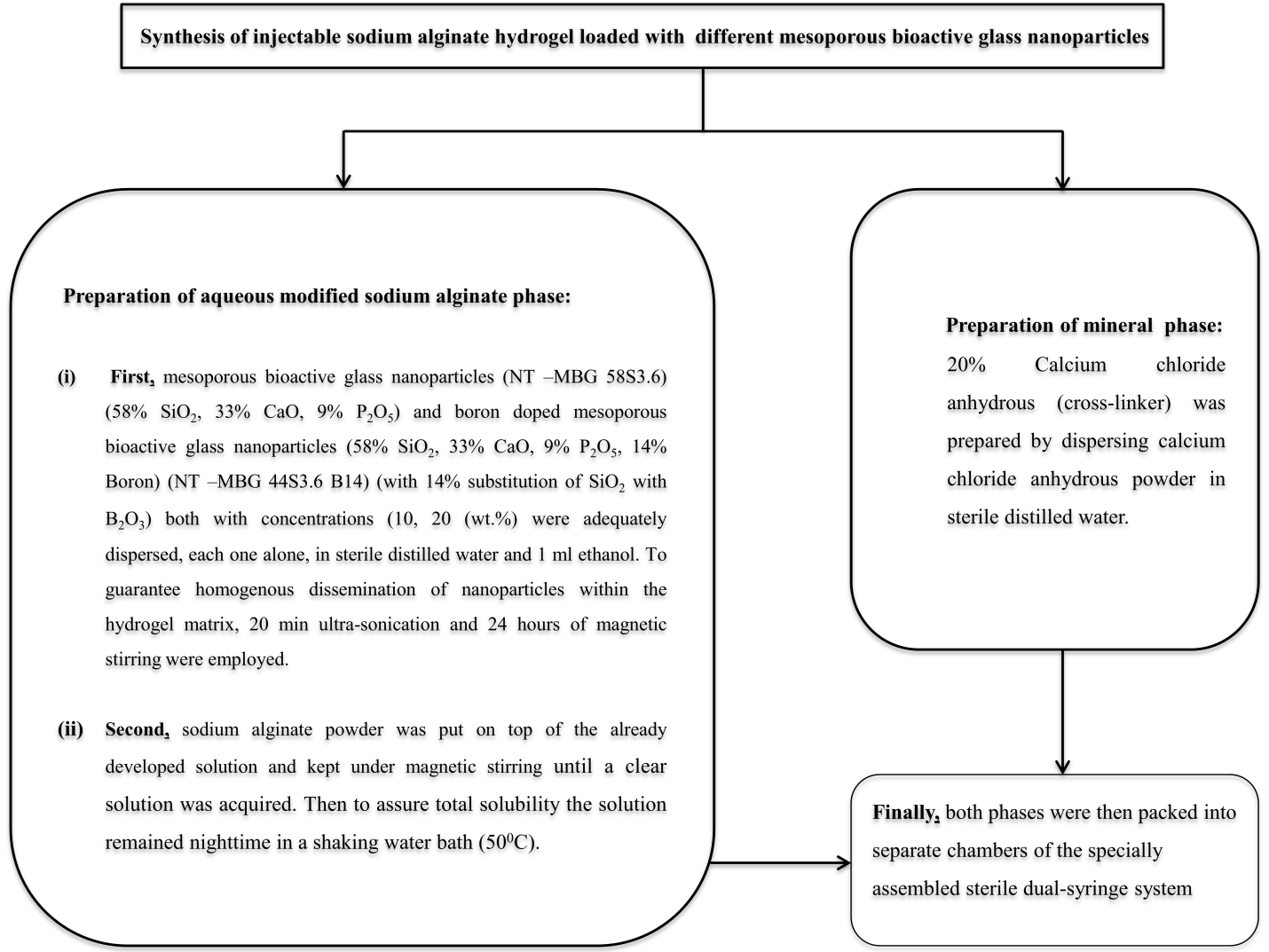


**Fig. S1** Schematic laboratory steps of preparing the injectable sodium alginate hydrogel loaded with different mesoporous bioactive glass nanoparticles in various concentrations (10 wt. %, 20 wt. %).


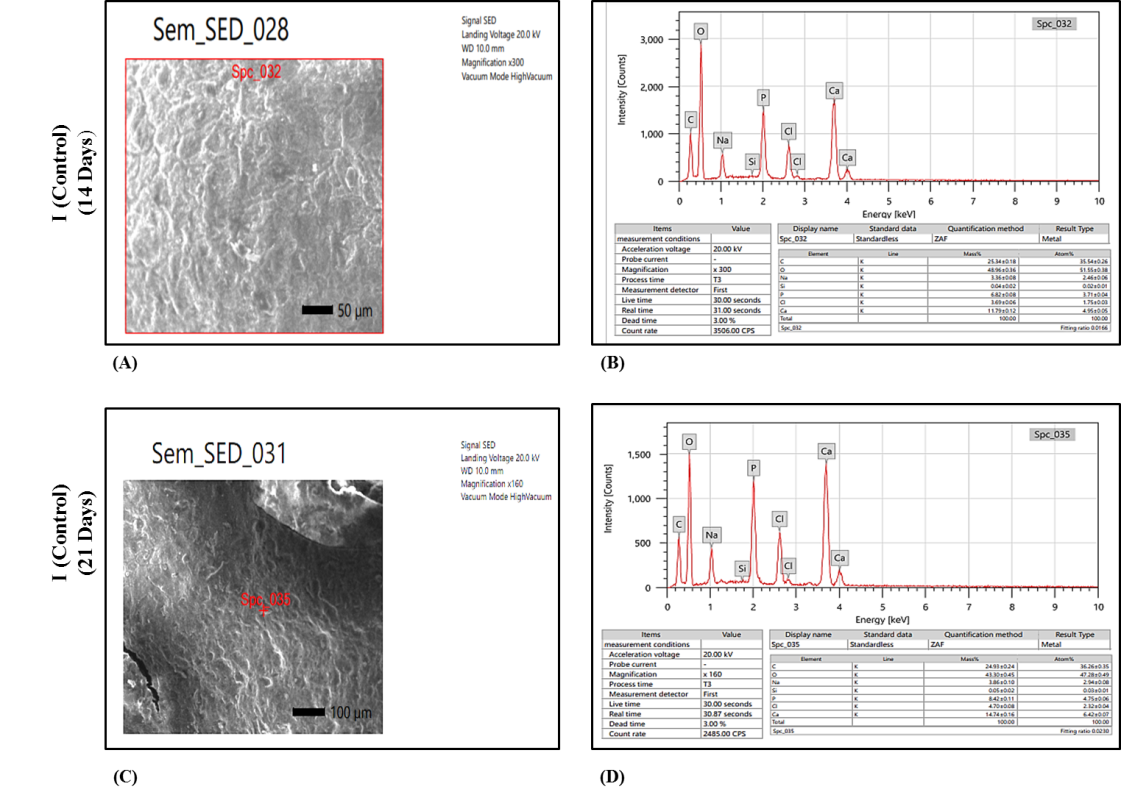


**Fig. S2** EDX spectra and Ca/P ratio of group I (Control) after soaking in SBF solution for 14 days (**A**, **B**) and 21 Days (**C**, **D**) displaying peaks for Ca and P (**A, C** magnification X300**,** X160; respectively).


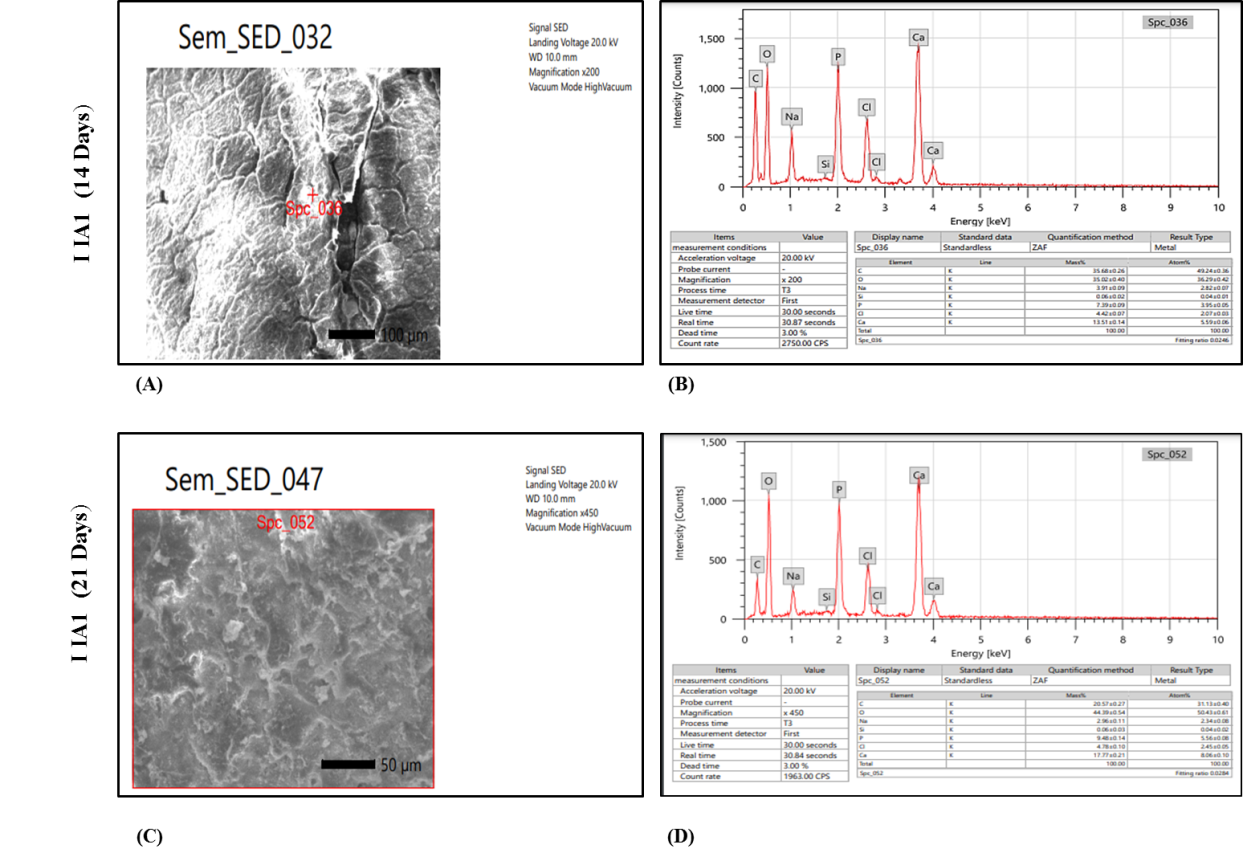


**Fig. S3** EDX spectra and Ca/P ratio of group IIA1 after soaking in SBF solution for 14 Days (**A**, **B**) and 21 Days (**C**, **D**) displaying peaks for Ca and P (**A, C** magnification X200**,** X450; respectively).


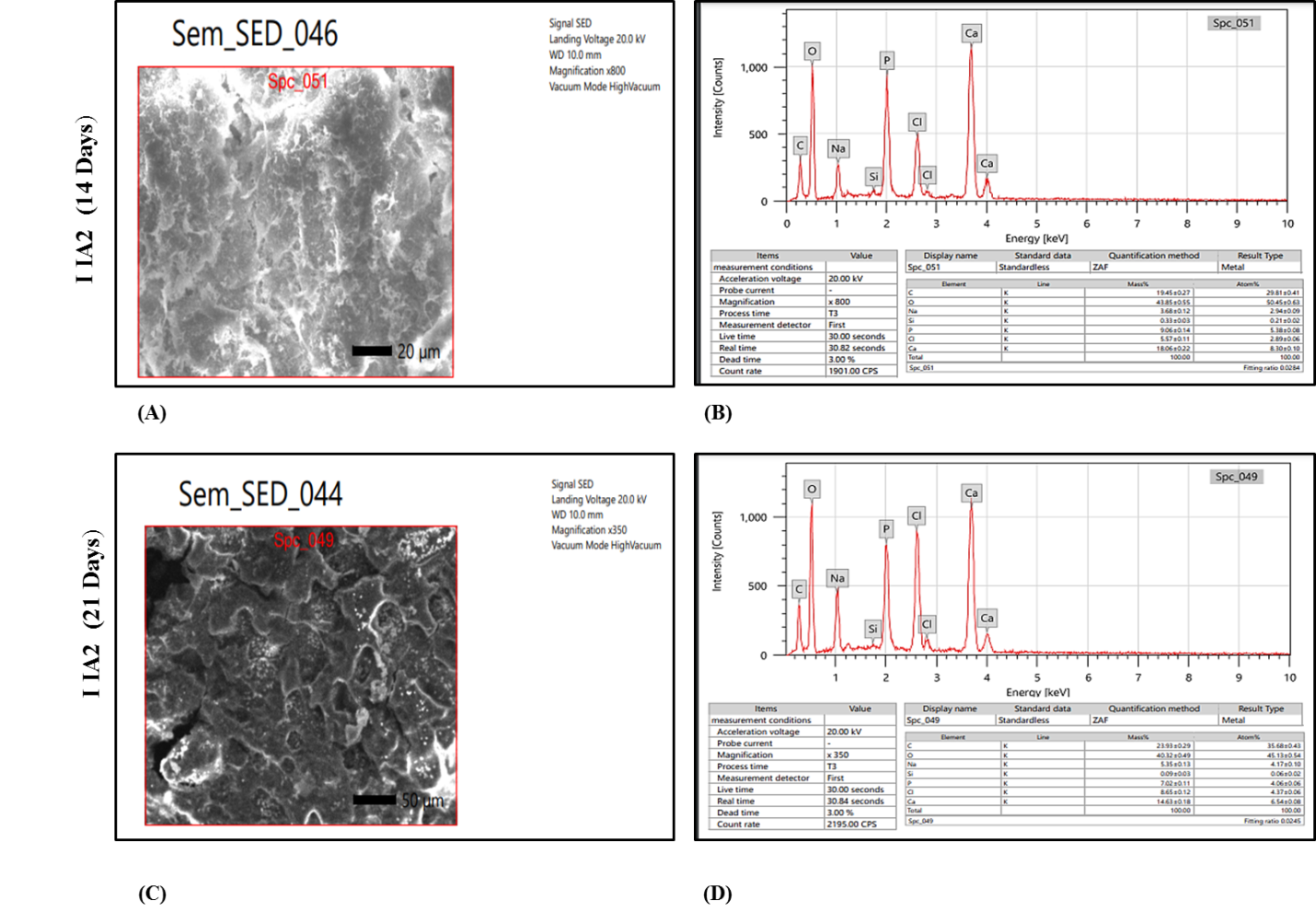


**Fig. S4** EDX spectra and Ca/P ratio of group IIA2 after soaking in SBF solution for 14 Days (**A**, **B**) and 21 Days (**C**, **D**) displaying peaks for Ca and P (**A, C** magnification X800**,** X350; respectively).


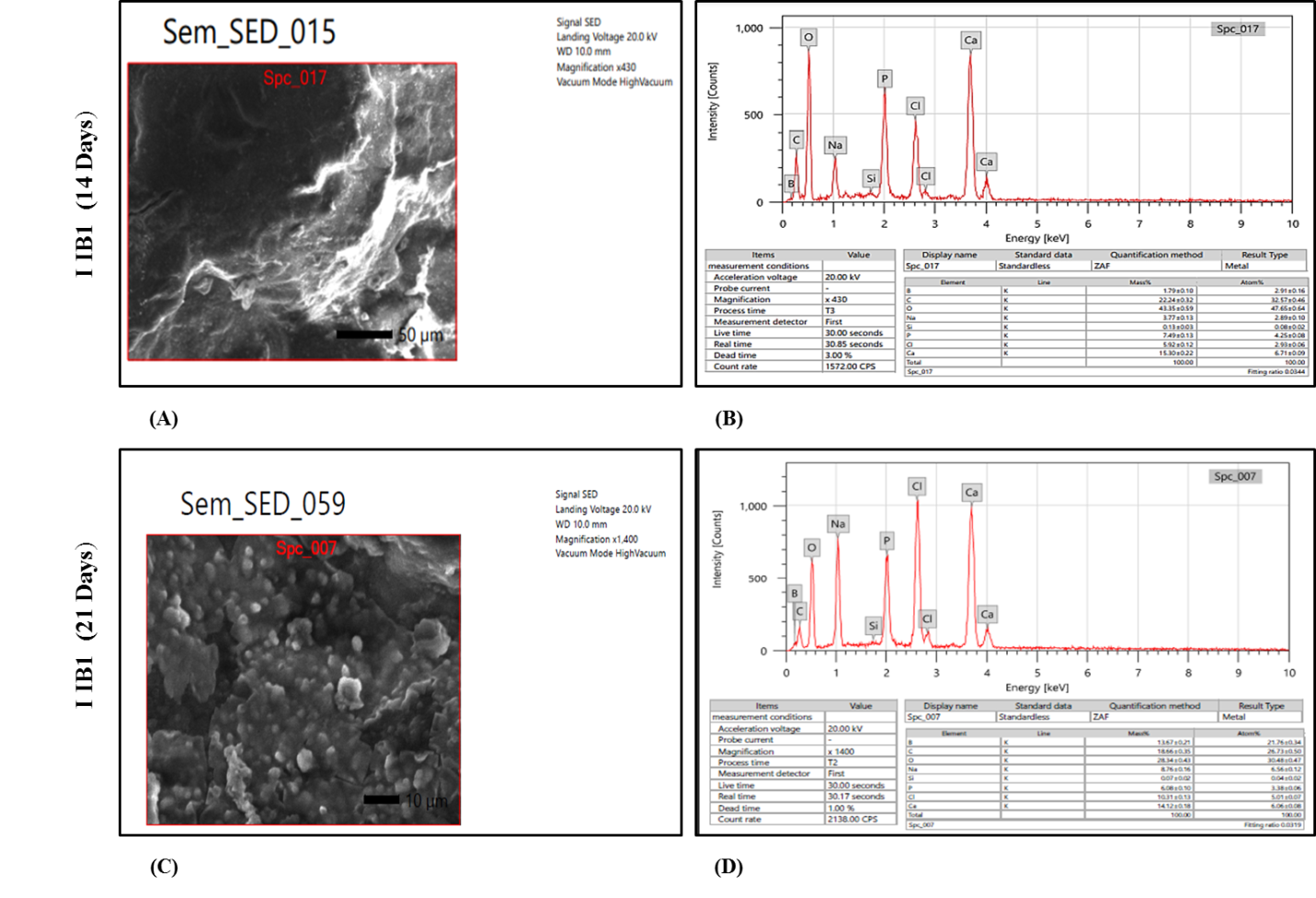


**Fig. S5** EDX spectra and Ca/P ratio of group IIB1 after soaking in SBF solution for 14 Days (**A**, **B**) and 21 Days (**C**, **D**) displaying peaks for Ca and P (**A, C** magnification X430 **,** X1400; respectively).


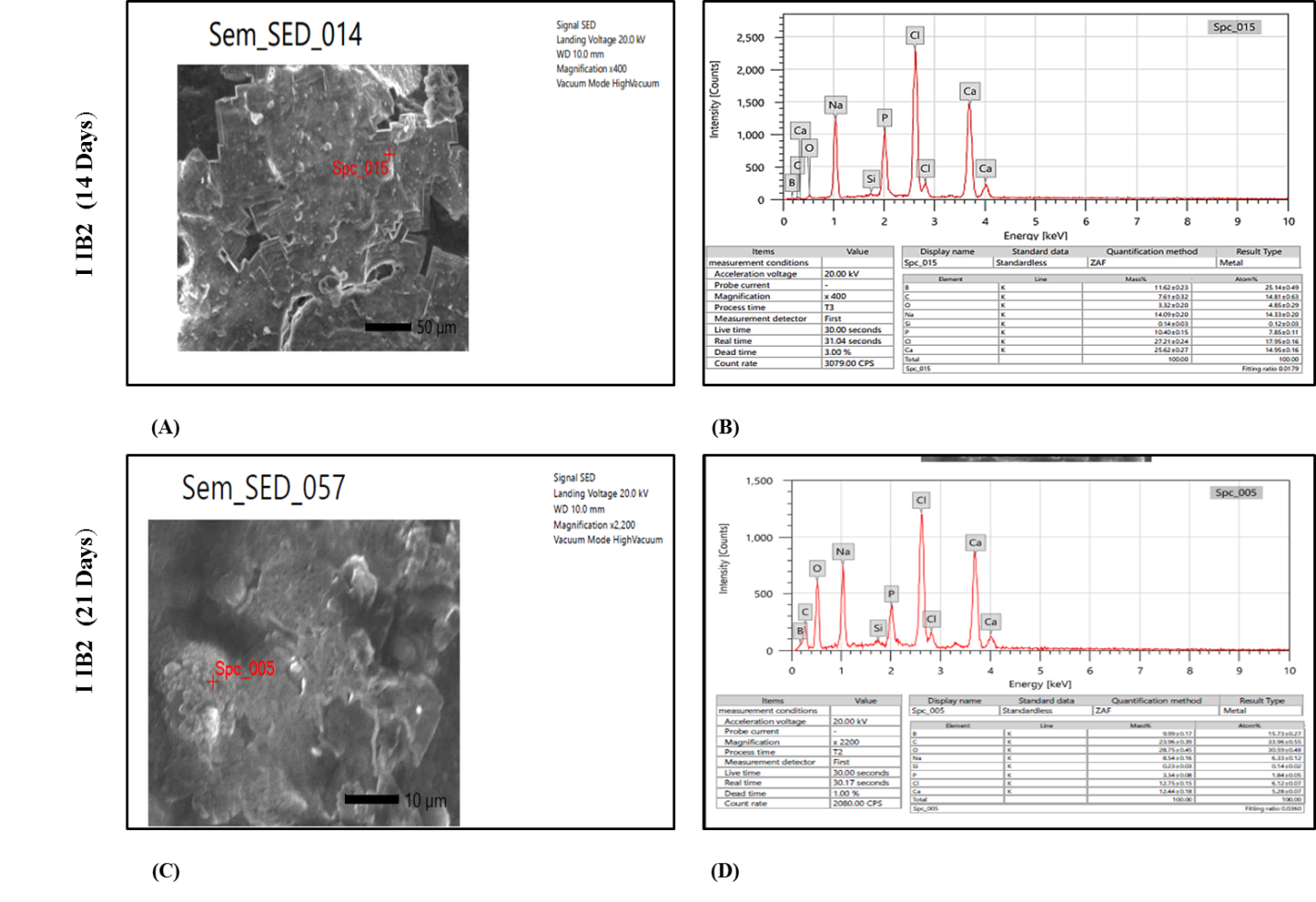


**Fig. S6** EDX spectra and Ca/P ratio of group IIB2 after soaking in SBF solution for 14 Days (**A**, **B**) and 21 Days (**C**, **D**) displaying peaks for Ca and P (**A, C** magnification X400**,** X2200; respectively).


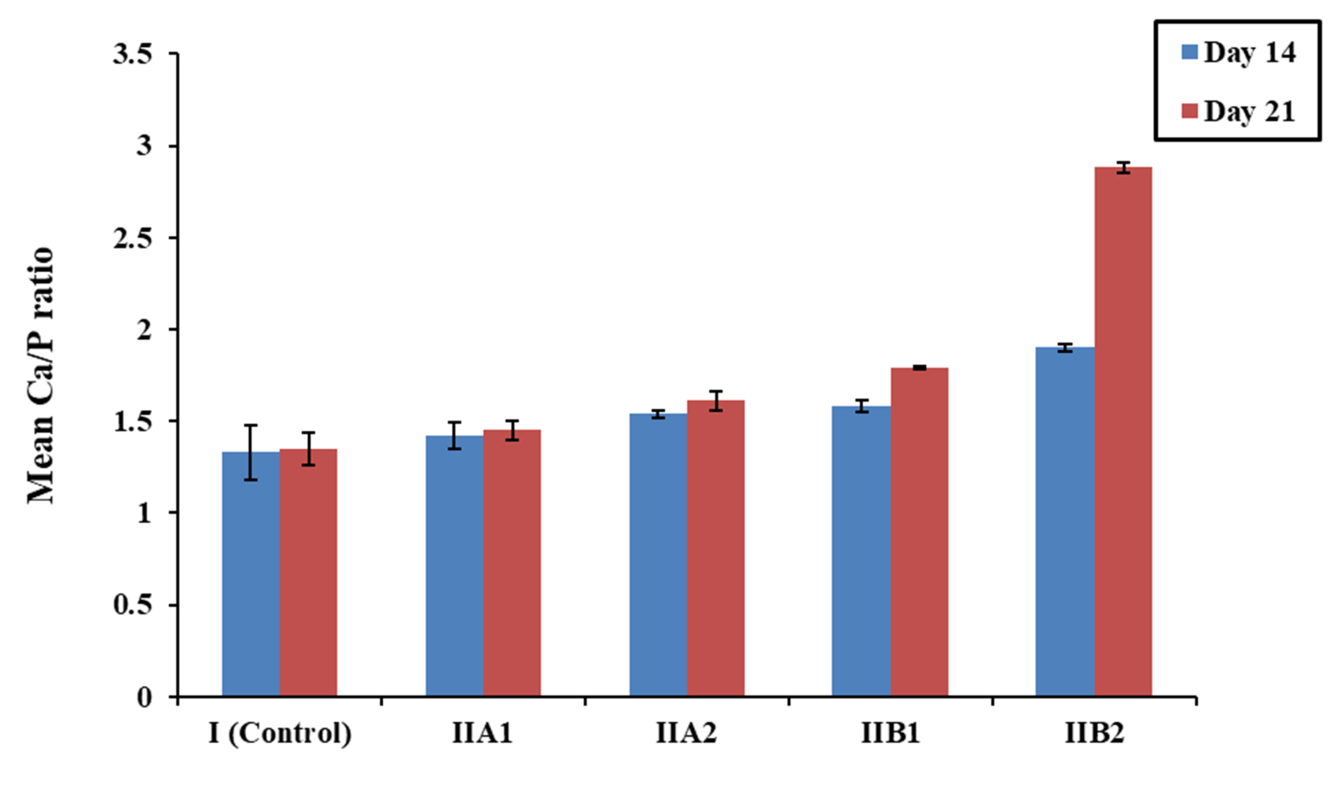


**Fig. S7** Mean Ca/P ratio of prepared specimens of all studied groups after 14 and 21 days of immersion
in SBF solution

**
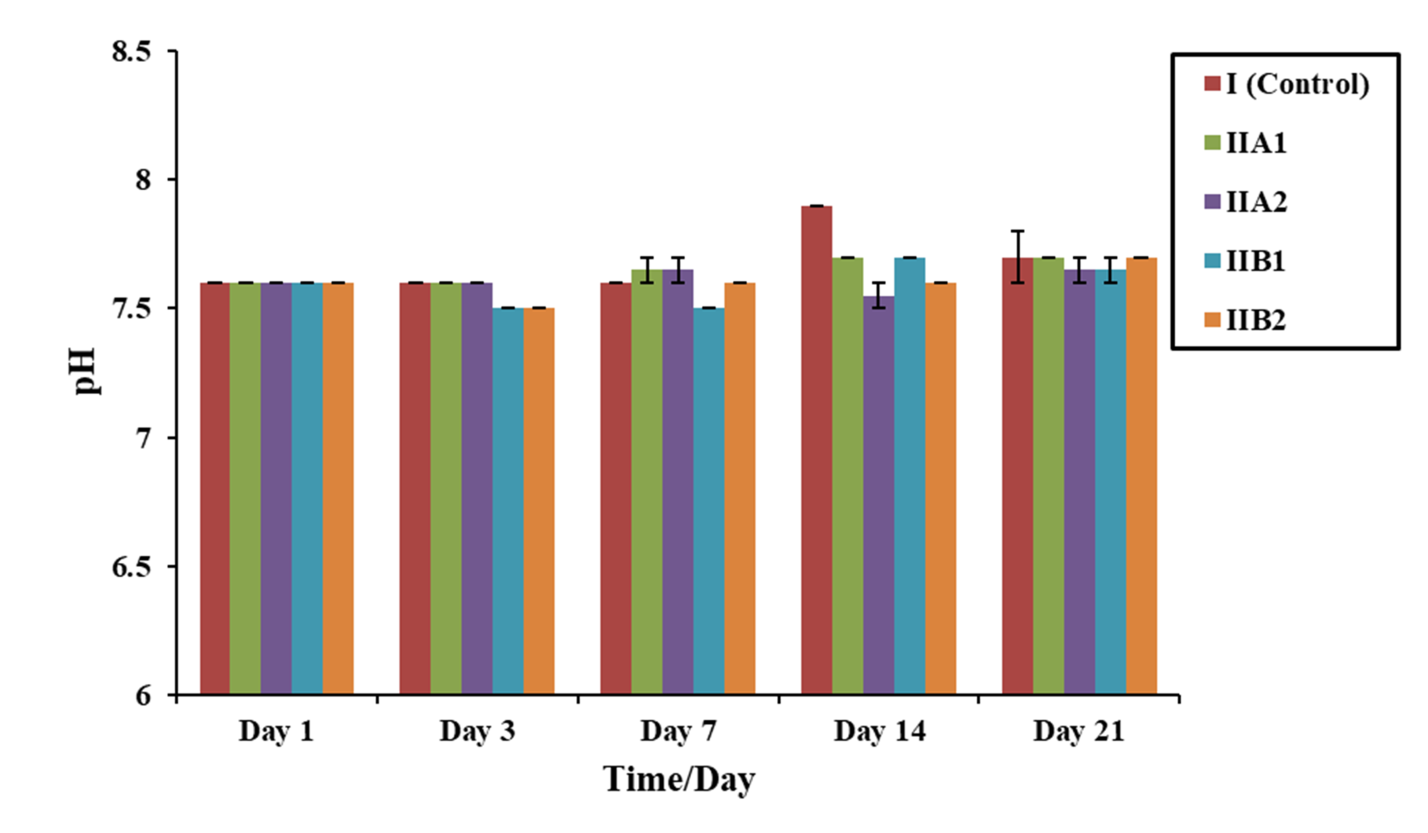
**

**Fig. S8** Alterations in SBF solution pH following contact with prepared hydrogel specimens after different time intervals.
